# Structure and engineering of the minimal type VI CRISPR-Cas13bt3

**DOI:** 10.1016/j.molcel.2022.08.001

**Published:** 2022-08-25

**Authors:** Ryoya Nakagawa, Soumya Kannan, Han Altae-Tran, Satoru N. Takeda, Atsuhiro Tomita, Hisato Hirano, Tsukasa Kusakizako, Tomohiro Nishizawa, Keitaro Yamashita, Feng Zhang, Hiroshi Nishimasu, Osamu Nureki

**Affiliations:** 1 Department of Biological Sciences, Graduate School of Science, The University of Tokyo, 7-3-1 Hongo, Bunkyo-ku, Tokyo 113-0033, Japan; 2 Broad Institute of MIT and Harvard, Cambridge, MA 02142, USA; 3 McGovern Institute for Brain Research at MIT, Massachusetts Institute of Technology, Cambridge, MA 02139, USA; 4 Department of Biological Engineering, Massachusetts Institute of Technology, Cambridge, MA 02139, USA; 5 Department of Brain and Cognitive Science, Massachusetts Institute of Technology, Cambridge, MA 02139, USA; 6 Graduate School of Medical Life Science, Yokohama City University, 1-7-29 Suehiro-cho, Tsurumi-ku, Yokohama, Kanagawa 230-0045, Japan; 7 MRC Laboratory of Molecular Biology, Francis Crick Avenue, Cambridge CB2 0QH, UK; 8 Structural Biology Division, Research Center for Advanced Science and Technology, The University of Tokyo, 4-6-1 Komaba, Meguro-ku, Tokyo 153-8904, Japan; 9 Department of Chemistry and Biotechnology, Graduate School of Engineering, The University of Tokyo, 7-3-1 Hongo, Bunkyo-ku, Tokyo 113-8656, Japan; 10 Inamori Research Institute for Science, 620 Suiginya-cho, Shimogyo-ku, Kyoto 600-8411, Japan

## Abstract

Type VI CRISPR-Cas13 effector enzymes catalyze RNA-guided RNA cleavage and have been harnessed for various technologies, such as RNA detection, targeting, and editing. Recent studies identified Cas13bt3 (also known as Cas13X.1) as a miniature Cas13 enzyme, which can be used for knockdown and editing of target transcripts in mammalian cells. However, the action mechanism of the compact Cas13bt3 remains unknown. Here, we report the structures of the Cas13bt3-guide RNA complex and the Cas13bt3-guide RNA-target RNA complex. The structures revealed how Cas13bt3 recognizes the guide RNA and its target RNA and provided insights into the activation mechanism of Cas13bt3, which is distinct from those of the other Cas13a/d enzymes. Furthermore, we rationally engineered enhanced Cas13bt3 variants and ultracompact RNA base editors. Overall, this study improves our mechanistic understanding of the CRISPR-Cas13 enzymes and paves the way for the development of efficient Cas13-mediated transcriptome modulation technologies.

## Graphical abstract

**Graphic abstract:**
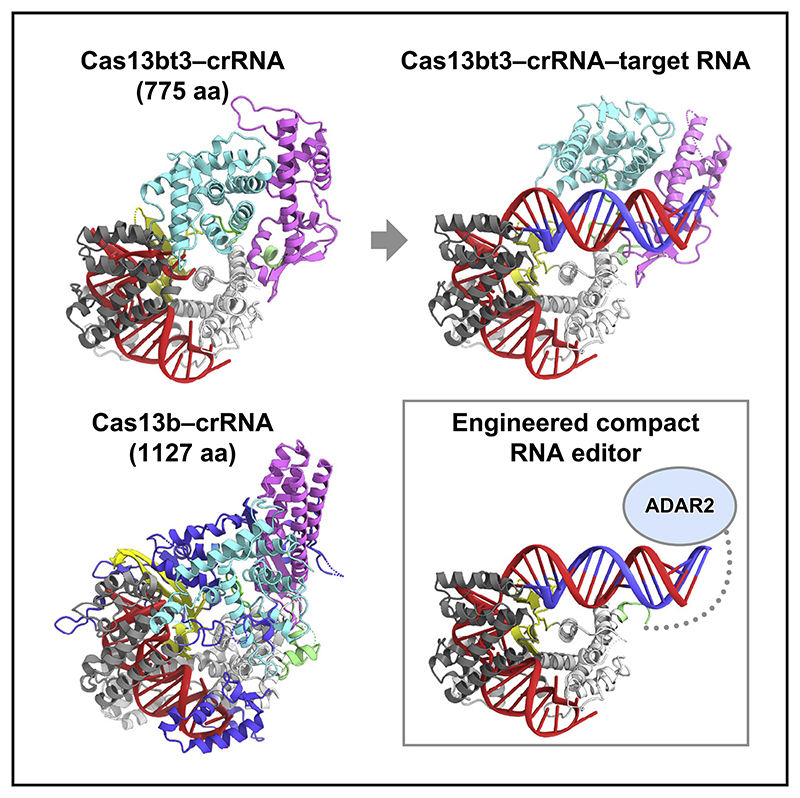

## Introduction

CRISPR-Cas systems in prokaryotes provide adaptive immunity against foreign nucleic acids and are divided into two classes (classes 1 and 2) (Hille et al., 2018; Makarova et al., 2020). In the class 1 systems, invading nucleic acids are degraded by effector complexes consisting of multiple Cas proteins and a CRISPR RNA (crRNA). In the class 2 systems, single multidomain Cas proteins associate with their guide RNA (crRNA or crRNA: tracrRNA [*trans*-activating crRNA] duplex) to form effector complexes responsible for target nucleic acid cleavage. The class 2 systems are further divided into three types, II, V, and VI, in which Cas9, Cas12, and Cas13 function as effector proteins, respectively. Cas9 associates with a crRNA and a tracrRNA and cleaves double-stranded DNA (dsDNA) targets, using the RuvC and HNH nuclease domains (Veits et al., 2012; Jinek et al., 2012). Although the Cas12 family proteins are functionally diverse, most of them cleave dsDNA targets using their single RuvC active site (Zetsche et al., 2015; Shmakov et al., 2015; Yan et al., 2019; Strecker et al., 2019; Makarova et al., 2020; Pausch et al., 2020). Since some Cas9 and Cas12 enzymes exhibit robust dsDNA cleavage activities in eukaryotic cells, they are used as powerful genomeediting tools (Cong et al., 2013; Zetsche et al., 2015).

Unlike Cas9 and Cas12, Cas13 is an RNA-guided RNA endonuclease containing two HEPN (higher eukaryotes and prokaryotes nucleotide-binding) nuclease domains (Abudayyeh et al., 2016; Smargon et al., 2017; Shmakov et al., 2017; Konermann et al., 2018; Yan et al., 2018). Cas13 associates with a crRNA and recognizes a single-stranded RNA (ssRNA) target complementary to the crRNA spacer sequence. Upon target RNA binding, Cas13 uses the HEPN nuclease domains and cleaves the target ssRNA in *cis* and bystander ssRNAs in *trans* (referred to as collateral cleavage) (Abudayyeh et al., 2016; East-Seletsky et al., 2016). Cas13 has a second RNase activity and processes its own precursor crRNAs (pre-crRNAs) to generate mature crRNAs in a HEPN-independent manner (East-Seletsky et al., 2016). The Cas13 family enzymes are divided into four subtypes (Cas13a–d) (Shmakov et al., 2017) and share the two HEPN domains (HEPN1 and HEPN2), but their domain arrangements and crRNA configurations are highly divergent. While the Cas13 enzymes commonly have C-terminal HEPN2 domains, Cas13a/c/d and Cas13b contain the HEPN1 domains at the center and N terminus of their primary structures, respectively. In addition, the crRNAs of Cas13a/c/d and Cas13b contain the direct repeat (DR) sequences upstream (5′ DR) and downstream (3′ DR) of the spacer sequence, respectively (Shmakov et al., 2017; Smargon et al., 2017).

Cas13a has been harnessed for RNA knockdown and detection (Abudayyeh et al., 2017; Gootenberg et al., 2017, 2018). In addition, the catalytically inactive Cas13b, fused to an adenosine or cytidine deaminase, mediates A-to-I or C-to-U substitutions on RNA targets and has thus been used for RNA editing (Cox et al., 2017; Abudayyeh et al., 2019). Compared with Cas9-mediated DNA editing (Komor et al., 2016), Cas13-mediated RNA editing has many advantages in terms of allowing the installation of temporary and non-heritable edits. However, the therapeutic delivery of Cas13-based RNA-editing systems remains challenging, since the sizes of the Cas13 genes identified so far exceed the packaging capacity of an adeno-associated virus (Wu et al., 2010).

Cas13bt3 (also known as Cas13X.1) was recently identified as a minimal Cas13 protein (775 residues) and used for RNA knockdown in mammalian cells (Xu et al., 2021; Kannan et al., 2022). Furthermore, the catalytically inactive Cas13bt3 (dCas13bt3), with the R84A/H89A/R739A/H744A mutations in the HEPN domains, fused to the deaminase domain of the hyperactive ADAR2 (adenosine deaminase acting on RNA 2) variant (ADAR2dd-E488Q), enabled A-to-I substitutions on target transcripts in mammalian cells, with high efficiency and specificity. Importantly, since Cas13bt3 is much smaller than other Cas13b enzymes (~1,000–1,400 residues), it can be readily packaged into therapeutic viral vectors.

Previous structural studies provided mechanistic insights into the crRNA-guided target RNA cleavage by diverse Cas13 enzymes. The crystal structures of the Cas13a-crRNA binary complexes, such as *Leptotrichia shahii* Cas13a (Liu et al., 2017a) and *Lachnospiraceae bacterium* Cas13a (Knott et al., 2017), revealed that Cas13a adopts a bilobed architecture consisting of a recognition (REC) lobe and a nuclease (NUC) lobe, in which the two HEPN domains form a composite active site. Subsequently, the crystal structure of the *Leptotrichia buccalis* Cas13a (LbuCas13a)-crRNA-target RNA ternary complex revealed that, upon target RNA binding, the HEPN domains undergo structural changes and adopt the cleavage-competent active conformation, thereby providing important information about the activation mechanisms of the Cas13a enzymes (Liu et al., 2017b). More recently, the cryoelectron microscopy (cryo-EM) structures of *Eubacterium siraeum* Cas13d (EsiCas13d) in distinct functional states illuminated the Cas13d-mediated RNA cleavage mechanism, which is similar to that of Cas13a (Zhang et al., 2018a). In addition, the crystal structures of Cas13b-crRNA binary complexes, such as *Bergeyella zoohelcum* Cas13b (BzoCas13b) (Zhang et al., 2018b) and *Prevotella buccae* Cas13b (PbuCas13b) (Slaymaker et al., 2019), revealed that although Cas13b contains the conserved HEPN domains, its overall structure is distinct from those of the Cas13a/d enzymes, consistent with the differences in their domain organizations and crRNA configurations. However, it remains unknown how the Cas13b family enzymes become activated upon target RNA binding, due to the lack of structural information about the Cas13b-crRNA-target RNA complex. Furthermore, the action mechanism of the miniature Cas13bt3 has not been elucidated.

Here, we report the crystal structure of Cas13bt3 in complex with a crRNA and the cryo-EM structure of Cas13bt3 in complex with a crRNA and its complementary target RNA. These structures, along with our mutational experiments and molecular dynamics (MD) simulations, provide mechanistic insights into crRNA recognition and target RNA cleavage by Cas13bt3, which are distinct from those of the previously described Cas13a/d enzymes. Furthermore, we rationally engineered Cas13bt3 variants with improved efficiency and specificity. Overall, these findings enhance our understanding of the diversity of the type VI CRISPR-Cas13 enzymes and establish a framework for Cas13-based therapeutic RNA targeting and editing.

## Results

### High-resolution crystal structure of the Cas13bt3-crRNA binary complex

**Figure 1:**
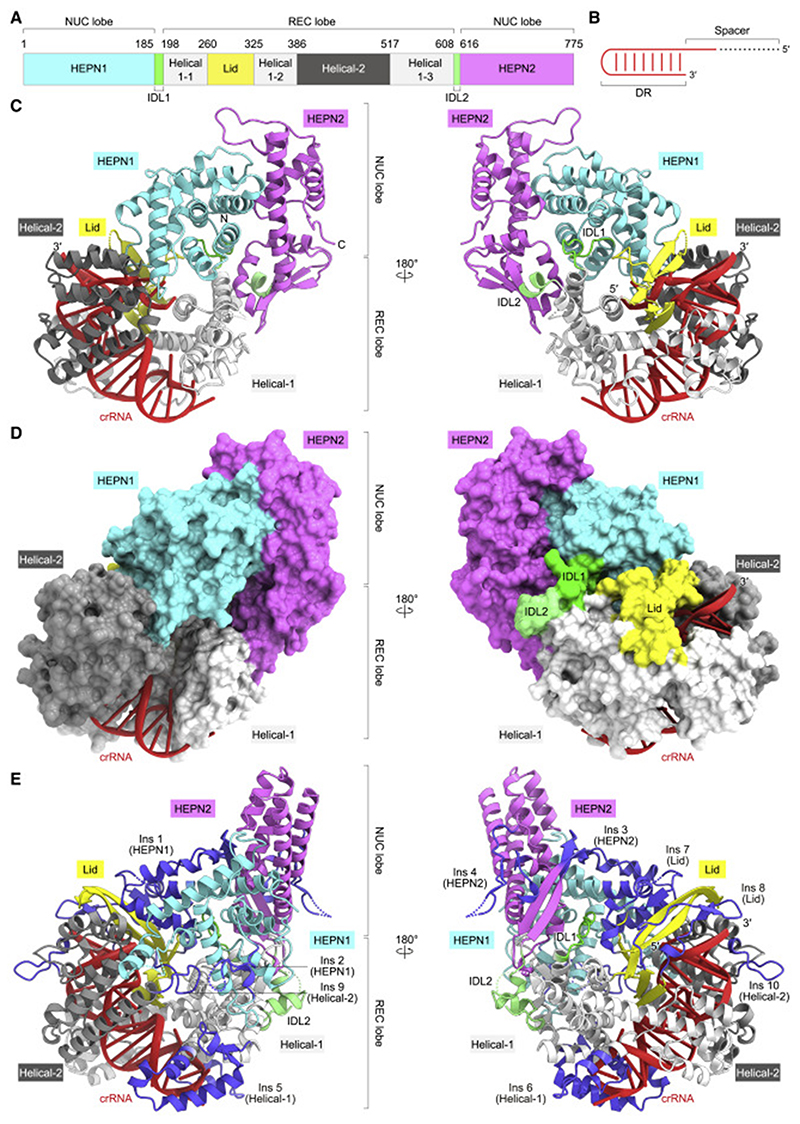
Crystal structure of the Cas13bt3-crRNA binary complex (A) Domain structure of Cas13bt3. IDL1, inter-domain linker 1; IDL2, inter-domain linker 2. (B) Diagram of the crRNA used for crystallization. (C and D) Ribbon (C) and surface (D) representations of the Cas13bt3-crRNA complex. In (C), disordered regions are indicated as dotted lines. (E) Structure of the PbuCas13b-crRNA complex (PDB: 4DTD). The PbuCas13b-specific insertions (Ins1–10) are highlighted in blue.

To understand how Cas13bt3 specifically assembles with its cognate crRNA, we solved the crystal structure of the catalytically inactive dCas13bt3 (R84A/H89A/R739A/H744A) in complex with a crRNA consisting of 36-nucleotide DR and 5-nucleotide spacer sequences (we used a short 5-nucleotide spacer to facilitate the crystallization), at 1.9-Å resolution (Figures 1A-1D; Table 1). The crystal structure revealed that Cas13bt3 adopts a bilobed architecture consisting of the REC and NUC lobes, with the crRNA DR anchored in the REC lobe (Figures 1C and 1D). The NUC lobe comprises the HEPN1 and HEPN2 domains, while the REC lobe consists of the Helical-1, Lid, and Helical-2 domains. The HEPN1 and HEPN2 domains are connected to the REC lobe by inter-domain linkers 1 (IDL1, residues 185–197) and 2 (IDL2, residues 608–615), respectively. Although Cas13bt3 (775 residues) is 352-residues smaller than PbuCas13b (1,127 residues) and shares only limited sequence identity (25%) with PbuCas13b, the overall structures of Cas13bt3 are similar to that of PbuCas13b (PDB: 4DTD, root-mean-square deviation [RMSD] of 4.4 Å for 420 equivalent Cα atoms) (Slaymaker et al., 2019) (Figures 1E and S1). A structural comparison of Cas13bt3 with PbuCas13b revealed the presence often major (>12 residues) insertions (Ins1-10) in PbuCas13b, as compared with Cas13bt3, thereby providing a structural explanation for the miniaturization of the Cas13bt3 protein scaffold, as described below.

The HEPN1 domain (residues 1–184) comprises nine α helices and two β strands, while the HEPN2 domain (residues 616–775) contains seven α helices and four β strands (Figure S1A). The HEPN1 and HEPN2 domains interact with each other to form an intramolecular dimer, in which the active site is composed of the catalytic residues (Arg84 and His89 in HEPN1 and Arg739 and His744 in HEPN2), although these residues are substituted with Ala in the present structure. The NUC lobe of Cas13bt3 structurally resembles that of PbuCas13b (RMSD of 2.6 Å for 233 equivalent Cα atoms) (Figures 1C, 1E, and S1A). Arg84, His89, Arg739, and His744 of Cas13bt3 are located at positions similar to those of the equivalent Arg156, His161, Arg1068, and His1073 of PbuCas13b, respectively (Figure S1A). The NUC lobe of PbuCas13b has four insertions (Ins 1–4), as compared with Cas13bt3 (Figures 1C, 1E, and S1A). The Helical-1 domain (residues 198–259, 325–385, and 517–607) of Cas13bt3 consists of three separate segments (Helical-1-1–3). Whereas the Helical-1-1 and Helical-1-2 domains of PbuCas13b are structurally similar to those of Cas13bt3, the Helical-1-3 domain of PbuCas13 has two insertions (Ins 5 and 6), which interact with the hairpin loop of the crRNA DR (Figures 1C, 1E, and S1B). The Lid domain (residues 260–324) of Cas13bt3 is composed of two tandemly arranged β sheets and interacts with the crRNA DR, whereas the Lid domain of PbuCas13b has two insertions (Ins 7 and 8) (Figures 1C, 1E, and S1C). The Helical-2 domain of Cas13bt3 (residues 386–516) consists of seven α helices and structurally resembles that of PbuCas13b, except for two long loops (Ins 9 and 10) (Figures 1C, 1E, and S1D). Our phylogenetic analysis revealed that Cas13bt proteins are not monophyletic relative to Cas13b proteins, suggesting that Cas13bt proteins evolved from larger ancestral Cas13b proteins through multiple deletions (Figure S1E). Taken together, the present structure explains the miniaturization of Cas13bt3.

### crRNA structure

**Figure 2:**
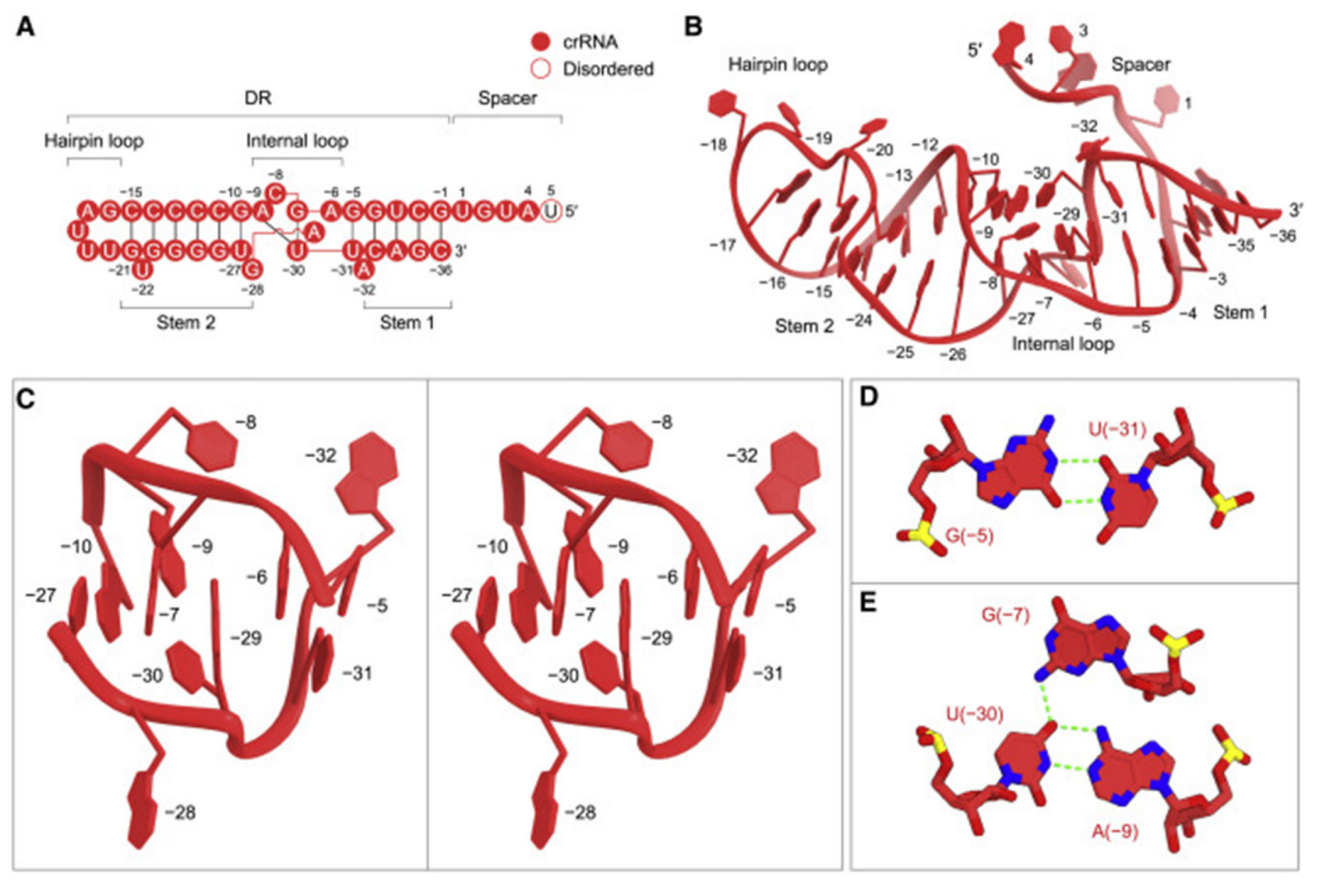
crRNA architecture (A) Sequence of the crRNA used for crystallization. (B)Structure of the crRNA in the binary complex. (C) Close-up view of the internal loop (stereo view). (D and E) The wobble base pair (D) and base triple (E) in the internal loop. Hydrogen bonds are depicted with green dashed lines.

The crRNA consists of the 5-nucleotide spacer (guide) segment and the 36-nucleotide DR region (Figures 2A and 2B).The DR region comprises stem 1 (G[–1]–G[–4] and C[–33]–C[–36]), an internal loop (G[–5]–A[–9] and G[–28]–A[–32]), stem 2 (G[–10]–C [–15] and G[–21]–U[–27]) and a hairpin loop (G[–16]–U[–20]) (Figures 2A and 2B). The electron density was less distinct for the spacer region, suggesting its flexibility in the Cas13bt3-crRNA binary complex structure (Figure S2A). Four nucleotides in the spacer (U1–A4) can be modeled in two different conformations, while U5 was not included in the model due to the lack of corresponding density (Figure S2B). As expected from the nucleotide sequence, stem 1 consists of four canonical Watson-Crick base pairs (G[–1]–C[–36]–G[–4]–C[–33]), and stem 2 contains a non-canonical G(–10)-U(–27) wobble base pair and five canonical base pairs (C[–11]–G[–26]–C[–14]–G[–23] and C[–15]–G [–21]), with U(–22) flipped out from the stem (Figures 2A and 2B). The internal loop of the Cas13bt3 crRNA adopts a distinct conformation from those of the PbuCas13b and BzCas13b crRNAs, consistent with their different sequences (Figures 2C, S2C, and S2D). U(–31) forms a non-canonical wobble base pair with G(–5) (Figure 2D), while U(–30) base pairs with A(–9) and G(–7) to form an A(–9)-U(–30) oG(–7) base triple (Figure 2E). Furthermore, C(–8), G(–28), and A(–32) are flipped out from the crRNA DR (Figures 2A and 2B).

### crRNA recognition

**Figure 3:**
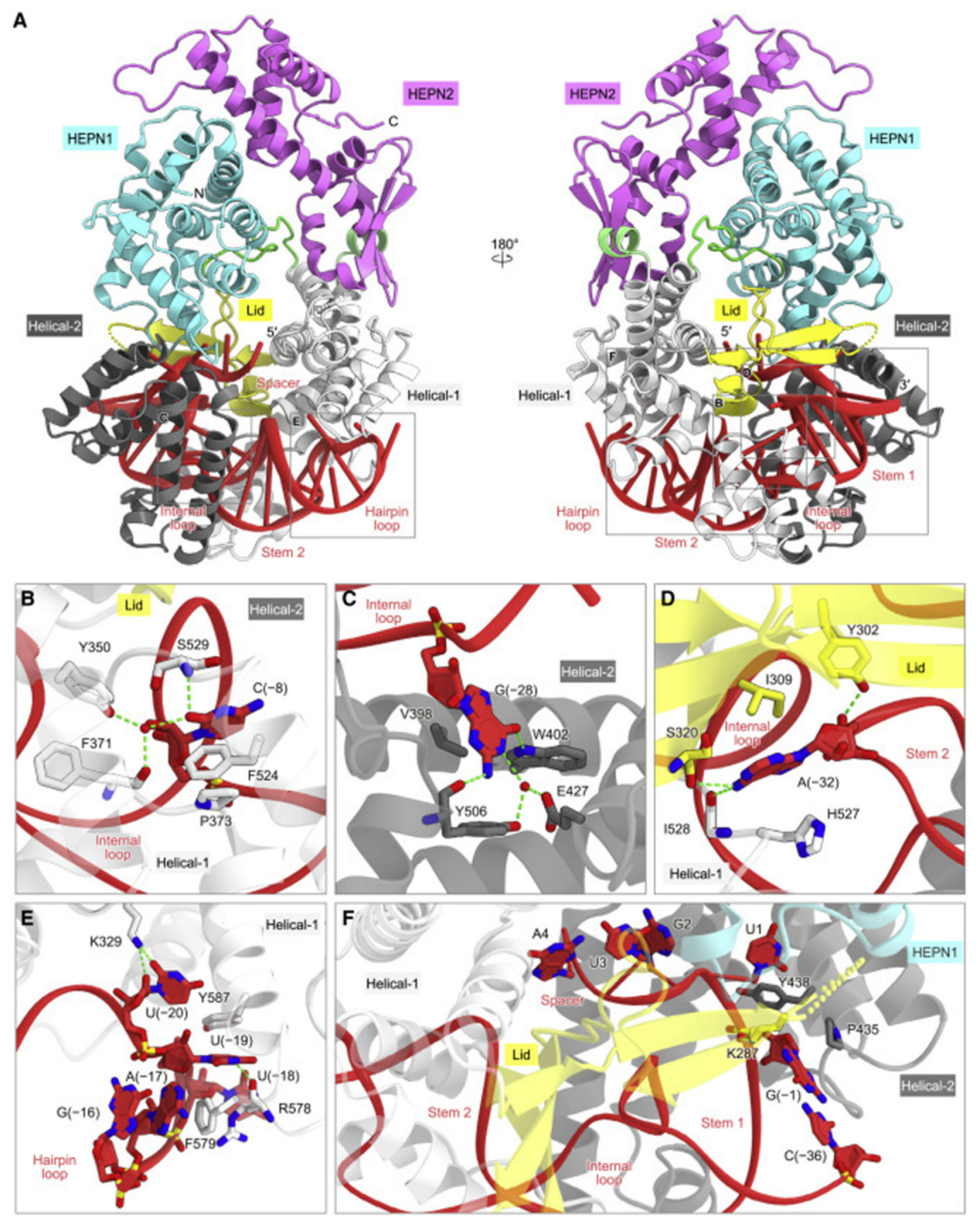
crRNA recognition (A) Recognition of the crRNA by Cas13bt3. (B–F) Recognition ofC(–8) (B), G(–28) (C), A(–32) (D), the hairpin loop (E), and the spacer-DRjunction (F). Hydrogen bonds are depicted with green dashed lines.

The crRNA DR is recognized by the Helical-1, Lid, and Helical-2 domains (Figures 3A and S3). The three flipped-out nucleotides, C(–8), G(–28), and A(–32), are extensively recognized by the protein. The C(–8) nucleobase is accommodated within a pocket composed of Pro373, Phe524, and Ser529, with the O2 atom forming a direct hydrogen bond with Ser529 and water-mediated hydrogen bonds with Tyr350 and Phe371 (Figure 3B). The G(–28) nucleobase forms a hydrogen-bonding network with Glu427, Trp402, and Tyr506 (Figure 3C). The A(–32) nucleobase is sandwiched between Ile309 and His527, with its N6 atom hydrogen bonding with the main-chain carbonyl groups of Ser320 and Ile528 (Figure 3D). The C(–8)G mutation in the crRNA reduced the Cas13bt3-mediated target RNA cleavage (Figures S2E and S2F), confirming the functional importance of the flipped-out C(–8) for the crRNA recognition. Moreover, A(–17), U(–19), and U(–20) in the hairpin loop are extensively recognized by the Helical-1 domain (Figure 3E). A(–17) stacks with Phe579, while U(–19) is sandwiched by Phe579 and Tyr587 and hydrogen bonds with the main-chain carbonyl groups of Arg578, and U(–20) forms a hydrogen bond with Lys329.

The crRNA is kinked at the DR-spacer junction, with the spacer region surrounded by the HEPN1, Helical-1, Lid, and Helical-2 domains (Figures 3A and 3F). U1 (the first nucleotide of the spacer) is recognized by His58, Arg115, His415, and Asn439, while G(–1) (the last nucleotide of the DR) forms hydrogen-bonding and hydrophobic interactions with Lys287 and Pro435, respectively (Figure 3F). The three nucleotides in the spacer (A2–G4) interact with the HEPN1, Lid, and Helical-1 domains within the protein molecule. These structural observations suggested that the DR-proximal region in the spacer cannot serve as a seed sequence that initiates base pairing with a target RNA. Our *in vitro* RNA cleavage assays demonstrated that double mismatches at the central region (mm3), but not the 5′ and 3′ regions (mm1 and mm4), of the spacer reduced the Cas13bt3-catalyzed RNA cleavage (Figure S2G). These results indicated that the seed sequence in Cas13bt3 crRNAs is around the central region, as in Cas13a crRNAs (Abudayyeh et al., 2016;Liu et al., 2017a). Our *in vitro* pre-crRNA processing experiments revealed that Cas13bt3 does not process its pre-crRNA, whereas PbuCas13b processes its pre-crRNA at the 3′ end (Figure S2H), as observed in previous studies (Smargon et al., 2017; Slaymaker et al., 2019). Consistently, the 3′ end of the Cas13bt3 crRNA does not interact with the protein and is exposed to the solvent in the present structure (Figure S2I). These results suggested that the Cas13bt3 pre-crRNA is processed by an unknown enzyme. Further studies will be required to elucidate the pre-crRNA processing mechanism of Cas13bt3.

### Cryo-EM structure of the Cas13bt3-crRNA-target RNA ternary complex

**Figure 4:**
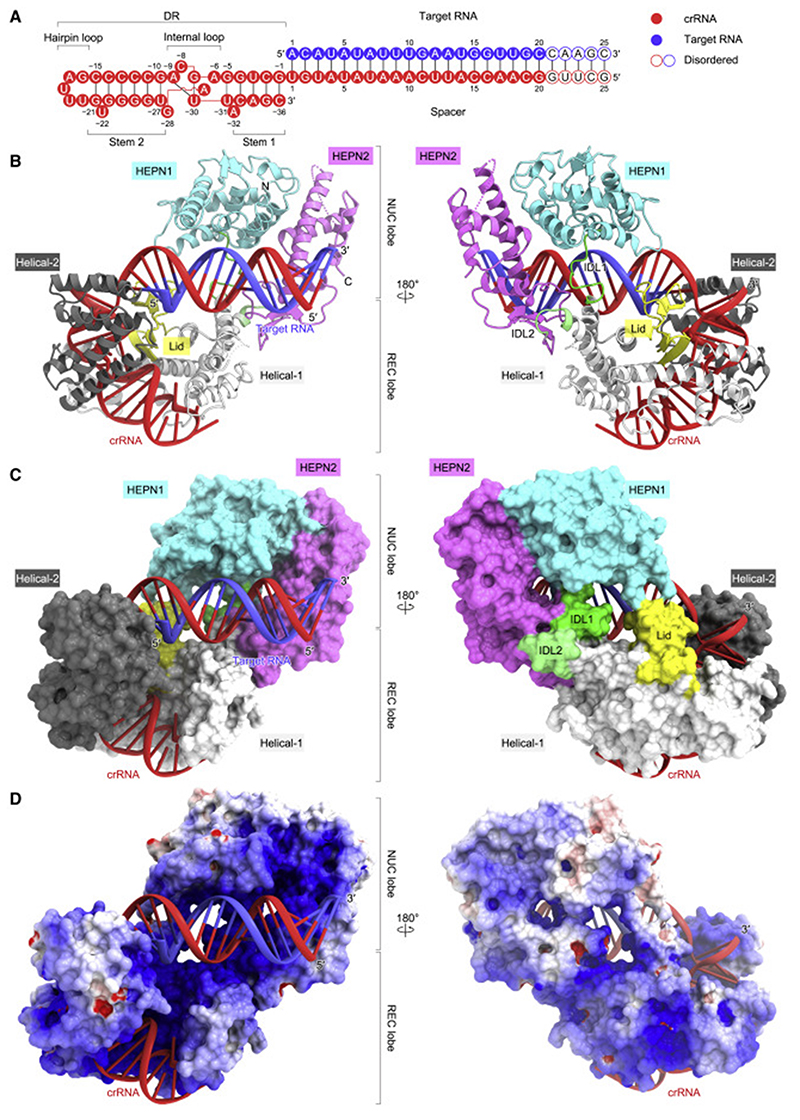
Cryo-EM structure of the Cas13bt3-crRNA-target RNA ternary complex (A) Sequences of the crRNA and target RNA used for the cryo-EM analysis. (B and C) Ribbon (B) and surface (C) representations of the Cas13bt3-crRNA-target RNA complex. In (B), disordered regions are indicated as dotted lines. (D) Electrostatic surface potential of Cas13bt3-crRNA-target RNA complex.

To clarify the crRNA-guided target RNA recognition and cleavage mechanisms of Cas13bt3, we determined the cryo-EM structure of dCas13bt3 in complex with a crRNA (61 nucleotides) and its complementary target RNA (25 nucleotides), at an overall resolution of 3.4 Å (Figures 4A, 4B, and S4A–S4C; Table 2). The density was less clear for the NUC lobe as compared with the REC lobe (Figure S4D), suggesting that the NUC lobe is relatively flexible in the ternary complex. Thus, we modeled the HEPN1/HEPN2 dimer structure in the binary complex into the density map as a single rigid body (Figure S4E), except for a flexible loop in the HEPN2 domain (residues 703–729, referred to as an active site loop [ASL]), which was not resolved in the density map. In the ternary complex, the crRNA DR (G[–1]–C[–36]) is anchored within the REC lobe, as in the binary complex (Figure 4B). The crRNA spacer (G20–U1) base pairs with the target RNA (A1–C20), while the terminal five base pairs (G21-C21–G25-C25) are disordered (Figure 4B). The crRNA-target RNA duplex is bound to the groove formed by the Helical-1, Lid, HEPN1, and HEPN2 domains (Figures 4C and 4D).

**Figure 5:**
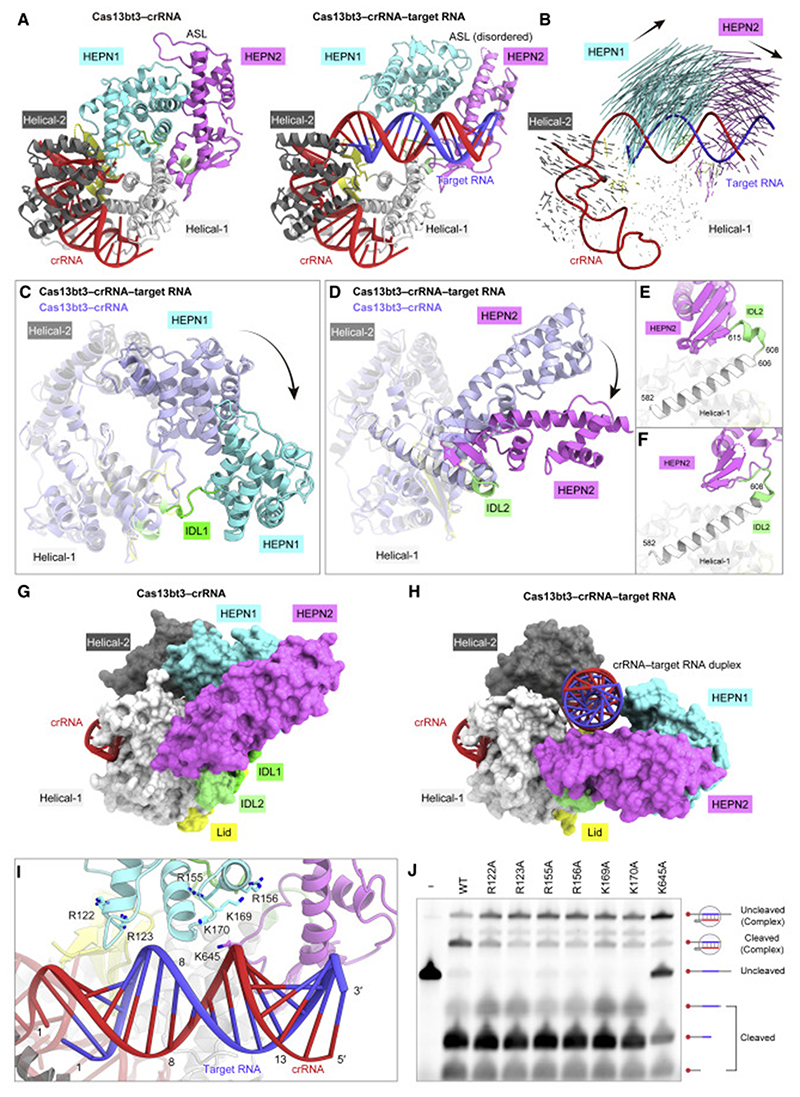
Structural differences between the Cas13bt3 binary and ternary complexes (A) Structural comparison of the Cas13bt3 binary (left) and ternary (right) complexes. (B) Structural transition between the binary and ternary complexes. Vectors indicate the transitions of equivalent Cα atoms between the binary and ternary complexes. (C and D) Structural changes in the HEPN1 (C) and HEPN2 (D) domains between the binary and ternary complexes. (E and F) Conformational transitions of IDL2 between the binary (E) and ternary (F) complexes. (G and H) Surface representations of the binary (G) and ternary (H) complexes. (I) Basic loop regions in the vicinity of the crRNA-target RNA duplex. (J) *In vitro* RNAc leavage activities of the wild-type and mutant Cas13bt3s. The 5^0^-Cy5-labeled target RNA, containing the 30-nt target sequence, was incubated with the Cas13bt3-crRNA complex for 60 min and then analyzed by denaturing urea PAGE.

A structural comparison between the binary and ternary complexes revealed notable differences in the arrangements of the HEPN1 and HEPN2 domains relative to the rest of the protein, although their individual domains are structurally similar (Figures 5A, 5B, and S5). Upon target RNA binding, the HEPN1 domain moves away from the REC lobe, with IDL1 serving as a pivot point (Figure 5C). In addition, IDL2 undergoes a local structural transition, allowing the rearrangement of the HEPN2 domain (Figure 5D). In the binary complex, residues 582–606 and 608–615 (IDL2) adopt long and short a helices, respectively (Figure 5E). In contrast, in the ternary complex, the short a helix is melted and residues 582–608 form a continuous a helix (Figure 5F). These structural rearrangements of the HEPN1 and HEPN2 domains result in the formation of a binding groove for the crRNA-target RNA duplex (Figures 5G and 5H). The crRNA-target RNA duplex is recognized by the Helical-1, Lid, HEPN1, and HEPN2 domains through interactions with its sugar-phosphate backbone (Figure S3). The central region of the crRNA-target RNA duplex is located close to three Arg/ Lys-rich loop regions in the HEPN1 (residues 122–134 and 153–175) and HEPN2 (residues 638–651) domains (Figure 5I). We individually mutated the conserved basic residues (Arg122, Arg123, Arg155, Arg156, Lys169, Lys170, and Lys645) in these regions and tested their effects on the RNA cleavage activities *in vitro*. The K645A mutation reduced the RNA cleavage (Figure 5J), confirming the functional importance of Lys645 for the crRNA-target RNA duplex recognition.

### RNA cleavage mechanism

**Figure 6:**
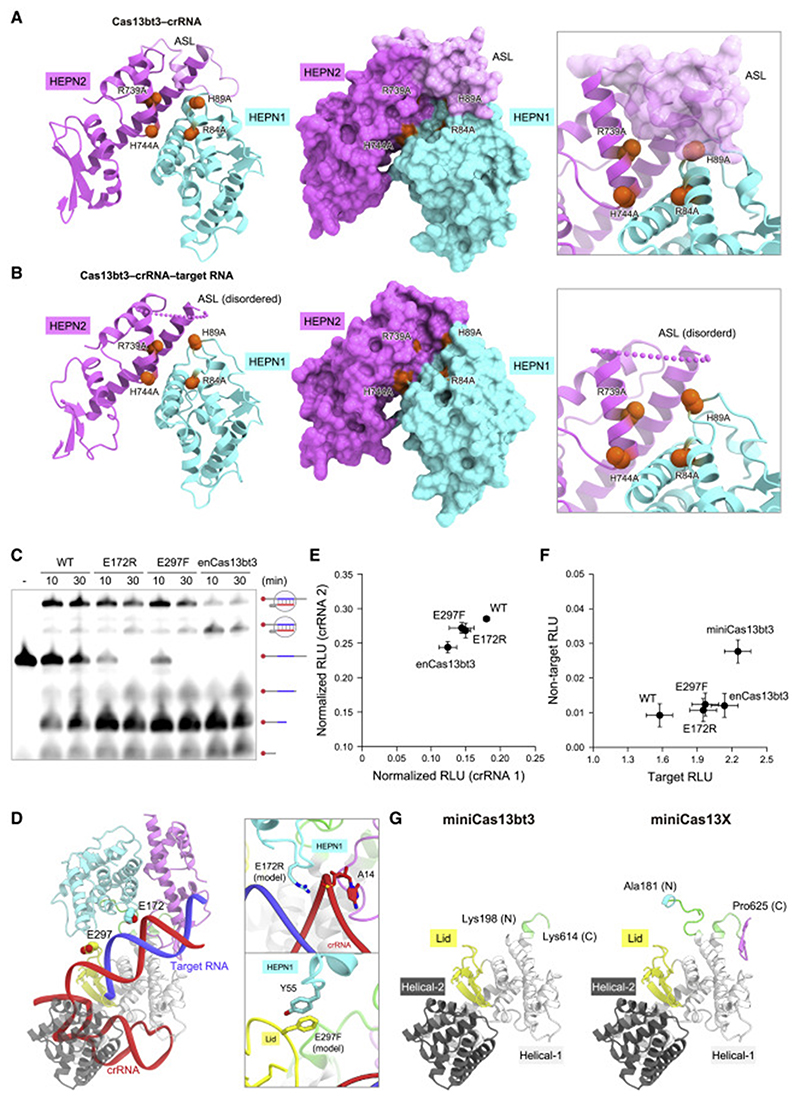
Activation mechanism and molecular engineering (A and B) HEPN active sites of the binary (A) and ternary (B) complexes. The catalytic residues are shown as red space-filling models. (C) *In vitro* RNA cleavage activities of the wild-type and mutant Cas13bt3s (E172R, E297F, and E172R/E297F [enCas13bt3]). The 5′-Cy5-labeled target RNA, containing the 30-nt target sequence, was incubated with the Cas13bt3-crRNA complex for 10 or 30 min and then analyzed by denaturing urea PAGE. (D) Locations of the E172R and E297F mutations in the ternary complex. (E)RNA knockdown activities of the wild-type and mutant Cas13bt3s in human cells. Data are normalized to a transfection control (without Cas13bt3 expression) and shown as mean ± SD (n = 4). (F) RNA-editing activities of the wild-type and mutant Cas13bt3s in human cells. RNA-editing efficiencies were evaluated based on the restoration of the luciferase activity from the *Cypridina* W85X luciferase reporter. A non-targeting crRNA was used as a proxy for evaluating the specificity. Data are shown as mean ± SD (n = 4). (G) Structural models of miniCas13bt3 and miniCas13X.

A structural comparison between the binary and ternary complexes revealed that, whereas the ASL is ordered and covers the HEPN1 active site in the binary complex (Figure 6A), it is disordered and the HEPN1 active site is accessible in the ternary complex (Figure 6B). Notably, the HEPN1 and HEPN2 domains interact with the Lid/Helical-2 and Helical-1 domains in the binary complex, whereas the HEPN domains do not interact with the REC lobe in the ternary complex (Figure 5A). These structural observations suggested that, due to the lack of interactions with the REC lobe, the HEPN domains have greater flexibility in the ternary complex as compared with the binary complex, thereby facilitating ASL dissociation from the HEPN active site.

To investigate the flexibilities of the binary and ternary complexes, we performed all-atom MD simulations and calculated the RMSF (root-mean-square fluctuation) values for the Cα atoms in the binary and ternary complexes during the 200-ns simulations. In two independent simulations, the NUC lobe exhibited higher RMSF values in the ternary complex as compared with the binary complex, whereas the REC lobe displayed comparable RMSF values in the two complexes (Figure S6A), suggesting the greater flexibility of the NUC lobe in the ternary complex. Importantly, the HEPN active site remained covered with the ASL in the binary complex during our simulations, whereas the HEPN active site was not occluded by the ASL in the ternary complex (Figure S6B).

These structural and functional analyses, together with our MD simulations, provided mechanistic insights into the Cas13bt3-mediated RNA-guided RNA cleavage. In the Cas13bt3-crRNA binary complex, the HEPN active site is covered by the ASL and thus inaccessible by ssRNA substrates. The Cas13bt3-crRNA complex recognizes a complementary ssRNA to form the crRNA-target RNA duplex, which facilitates the rearrangement of the HEPN domains. In the Cas13bt3-crRNA-target RNA ternary complex, the HEPN domains move away from the REC lobe, and thus, the ASL becomes more flexible and dissociates from the HEPN active site, thereby allowing the access of ssRNA substrates to the catalytic residues. The ASLs in the PbuCas13b and BzoCas13b binary complexes cover their HEPN active sites, as in the Cas13bt3 binary complex (Figure S6C), suggesting that the activation mechanisms are conserved among the Cas13b family enzymes.

### Structure-guided engineering

To improve the utility of Cas13bt3 in RNA targeting and editing, we sought to engineer a Cas13bt3 variant with better RNA-binding ability. We selected 17 residues and designed 28 Cas13bt3 mutants with amino acid substitutions that may provide new interactions within the Cas13bt3-crRNA-target RNA complex (Figure S6D). Among the 28 mutants, the E172R and E297F mutants exhibited higher RNA cleavage activities, as compared with the wild-type Cas13bt3 (Figure 6C). The E172R/E297F double mutation further enhanced the RNA cleavage activity (Figure 6C). The E172R mutation is located at the HEPN1 domain and could establish a new interaction with the crRNA backbone phosphate, while the E297F mutation is in the Lid domain and could form a stacking interaction with Tyr55 in the HEPN1 domain (Figure 6D). We will hereafter refer to the E172R/E297F variant as the enhanced Cas13bt3 (enCas13bt3).

To evaluate the RNA-targeting ability of enCas13bt3, we measured RNA knockdown by the wild-type Cas13bt3 and en-Cas13bt3 in HEK293FT cells, using two different crRNAs (crRNAs 1 and 2) targeting a luciferase reporter gene. en-Cas13bt3 exhibited higher knockdown efficiency, as compared with the wild-type Cas13bt3 (Figure 6E). To examine the applicability of enCas13bt3 to RNA editing, we fused the catalytically inactive mutant of Cas13bt3 or enCas13bt3 with ADAR2dd to create Cas13bt3-REPAIR and enCas13bt3-REPAIR, respectively, and then measured their A-to-I RNA-editing efficiencies, using a crRNA targeting an artificial UAG stop codon in a luciferase reporter gene (A-to-I editing should revert the artificial UAG stop codon to a UGG codon for Trp85). enCas13bt3-REPAIR mediated the RNA editing with improved efficiency and similar specificity, as compared with Cas13bt3-REPAIR (Figure 6F). These results suggested the utility of enCas13bt3 for RNA targeting and editing in human cells.

A recent study reported that a Cas13bt3 variant (referred to as miniCas13X), lacking the HEPN1 (residues 1–180) and HEPN2 domains (residues 626–775), functions as a compact RNA-targeting platform and can be used for RNA editing (Xu et al., 2021), consistent with our structural finding that the HEPN domains of Cas13bt3 are not involved in the crRNA recognition. Our Cas13bt3 structure suggested that 28 residues (residues 181–197 in HEPN1 and residues 615–625 in HEPN2) could be further truncated from the miniCas13X variant (Figure 6G). To test this idea, we fused dCas13bt3, lacking residues 1–197 and 615–775, with ADAR2dd to create miniCas13bt3-REPAIR, and measured its RNA-editing activity in human cells. Notably, miniCas13bt3-REPAIR exhibited improved RNA-editing activity, as compared with Cas13bt3-REPAIR and enCas13bt3-REPAIR (Figure 6F). These results demonstrated that miniCas13bt3 functions as an ultracompact RNA-targeting platform compatible with RNA editing. PbuCas13 and BzoCas13b share similar overall structures with Cas13bt3, suggesting that other Cas13b proteins lacking the HEPN domains could also serve as compact RNA-targeting platforms.

## Discussion

In this study, we determined the crystal structure of the Cas13bt3-crRNA binary complex, revealing how Cas13bt3 recognizes its cognate crRNA to form the compact effector complex. A structural comparison of Cas13bt3 with other Cas13b enzymes highlighted their architectural conservation and divergence, providing a structural explanation for the miniaturization of the Cas13bt3 scaffold. We also determined the cryo-EM structure of the Cas13bt3-crRNA-target RNA ternary complex, which represents the first ternary complex structure of the Cas13b family enzymes. The present Cas13bt3 structures, together with our functional and computational analyses, have provided insights into the Cas13b-mediated RNA cleavage mechanisms.

**Figure 7:**
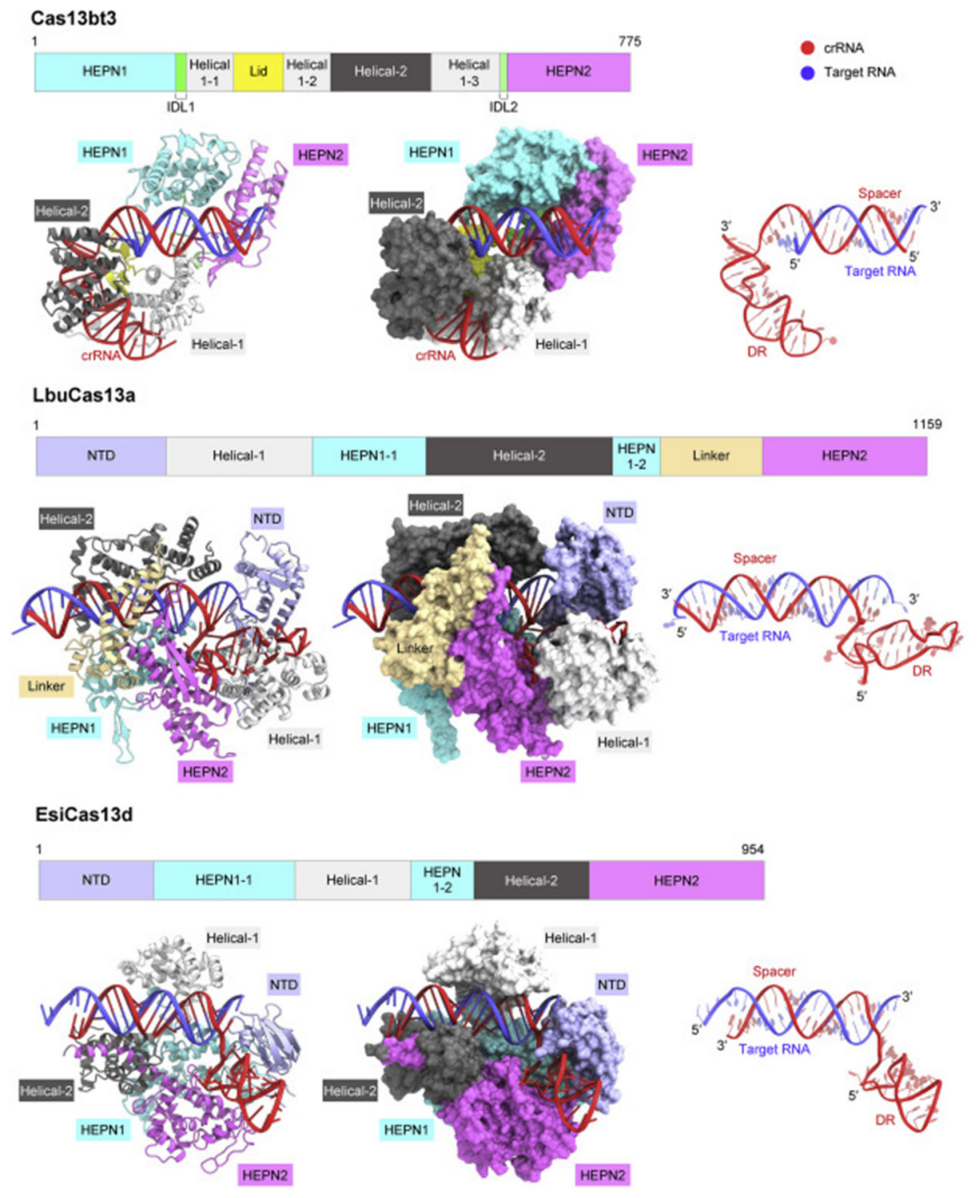
Comparison of diverse type VI CRISPR-Cas13 enzymes The ternary complex structures of Cas13bt3, LbuCas13a (Liu et al., 2017) (PDB: 5XWP), and EsiCas13d (Zhang et al., 2018) (PDB: 6E9F) are shown.

A structural comparison of Cas13bt3 with Cas13a (Liu et al., 2017b) and Cas13d (Zhang et al., 2018b) indicated notable differences in the activation mechanisms between Cas13b and Cas13a/d (Figures 7 and S7A–S7C). In the Cas13a/d binary complex structures, the HEPN active sites adopt inactive conformations, in which the catalytic residues are farther apart from each other (Figure S7D). In contrast, in the Cas13a/d ternary complex structures, the HEPN active sites adopt active conformations, in which the catalytic residues are closer to each other (Figure S7D). Thus, the HEPN active sites in Cas13a/d undergo substantial structural changes upon crRNA-target RNA duplex formation, thereby leading to the catalytically competent states. The current structures indicated that the HEPN active sites of Cas13bt3 adopt similar configurations regardless of the target RNA binding (Figure S7D). Unlike Cas13a/d, Cas13bt3 undergoes a rearrangement of the HEPN domains upon target RNA binding (Figure S7A–S7C). Consequently, the ASL may become more flexible and dissociate from the HEPN active site, thereby providing access of the catalytic residues to ssRNA targets (Figure S7D). Furthermore, a structural comparison of Cas13bt3 with Cas13a/d revealed differences in their mechanisms of crRNA-target RNA duplex recognition. The RNA duplex is accommodated within a central channel and not readily accessible from the solvent in the Cas13a/d structures, whereas it is bound to a surface groove in the Cas13bt3 structure and is largely exposed to the solvent (Figure 7). These structural differences suggest that the RNA duplex in the Cas13b complex is more readily accessible by the ADARdd fused to Cas13, as compared with those in the Cas13a/d complexes, thereby explaining why the Cas13b family proteins have been widely used for RNA-editing applications.

We rationally engineered the enCas13bt3 variant, which contains the E172R and E297F substitutions and exhibits enhanced RNA-targeting activity. The Cas13bt3 structure suggested that Arg172 (E172R) reinforces the interaction with the crRNA backbone phosphate, similar to substitutions that enhance protein-DNA interactions in the Cas9 variants (Hirano et al., 2016; Nishimasu et al., 2018). These results demonstrated that the reinforcement of protein-nucleic acid interactions is a general strategy to improve the nuclease activities of the diverse CRISPR-Cas enzymes, including Cas9 and Cas13. Unlike Arg172, Phe297 (E297F) likely reinforces the inter-domain interaction and stabilizes the active conformation of Cas13bt3. This finding suggests that the stabilization of the active conformation can be an alternative strategy to improve the nuclease activities of the CRISPR-Cas enzymes. Given that the HNH nuclease domain of Cas9 adopts both inactive and active conformations during target DNA cleavage (Shibata et al., 2017; Zhu et al.,2019), amino acid substitutions that stabilize the active conformation of the HNH domain might enhance the DNA cleavage activity of Cas9.

In conclusion, these Cas13bt3 structures provided mechanistic insights into the miniaturization of Cas13bt3 and the RNA cleavage by the Cas13b family enzymes. Furthermore, we rationally engineered Cas13bt3 variants, such as en-Cas13bt3 and miniCas13bt3-REPAIR. Our findings expand the understanding of diverse type VI CRISPR-Cas13 enzymes and will facilitate the development of efficient RNA-editing technologies.

### Limitations of the study

Our structural, functional, and computational data suggested that the activation mechanism of Cas13bt3 is distinct from those of the Cas13a/Cas13d enzymes. Nonetheless, the NUC lobe in the ternary complex structure was not well resolved in the density map, probably due to its flexibility. Therefore, it will be important to investigate the functional role of the ASL for the Cas13bt3 activation in the future. In addition, structural analyses of other Cas13b enzymes bound to target RNAs will be required to understand the activation mechanism of Cas13b family enzymes.

## Star Methods

### Key Resources Table

**Table:** 

REAGENT or RESOURCE	SOURCE	IDENTIFIER
**Chemicals, peptides, and recombinant proteins**		
Cas13bt3	Kannan et al, 2022	N/A
Cas13bt3, various mutants	This paper	N/A
PbuCas13b	Slaymaker et al., 2019	N/A
**Deposited data**		
Cas13bt3 binary complex coordinates	This paper	PDB: 7VTI
Cas13bt3 ternary complex coordinates	This paper	PDB: 7VTN
Cas13bt3 ternary complex EM map	This paper	EMDB: EMD-32118
Raw movies of the cryo-EM dataset Experimental models: Cell lines	This paper	EMPIAR ID: EMPIAR-11110
**Experimental models: Cell lines**		
*E. coli* Mach1	Thermo Fisher Scientific	C862003
*E. coli* Rosetta 2 (DE3)	Novagen	71397
HEK293T cells	American Type Culture Collection (ATCC)	N/A
**Oligonucleotides**		
DNA primers	This paper	Table S1
DNA oligos	This paper	Table S1
Cas13bt3 crRNAs	Kannan et al., 2022	Table S1
Cas13bt3 crRNA, and various mutants	This paper	Table S1
Cas13bt3 pre-crRNA	This paper	Table S1
PbuCas13b crRNA	Slaymaker et al., 2019	Table S1
PbuCas13b pre-crRNA	This paper	Table S1
**Recombinant DNA**		
pE-SUMO-mH6-Cas13bt3	This paper	https://benchling.com/s/seq-zHRTKg4EdlCE5SxK5jgH?m=slm-dSpLHltNtiFWbDvfpZDS
pE-SUMO-mH6-PbuCas13b	This paper	https://benchling.com/s/seqISu0dXt1vJgYst3qF5RX?m=slm-8Spih3qC6E4XES4nJwQl
U6-BpiI-Cas13bt3-DR	Kannan et al., 2022	N/A
CMV-HIVNES-GS-Cas13bt3	Kannan et al., 2022	N/A
CMV-HIVNES-GS-dCas13bt3-(GGS)2-huADAR2dd (E488Q)	Kannan et al., 2022	N/A
**Software and algorithms**		
KAMO	Yamashita et al., 2018	https://github.com/keitaroyam/yamtbx/blob/master/doc/kamo-en.md
XDS	Kabsch et al., 2010	https://xds.mr.mpg.de
SHELXC/D/E	Sheldrick, 2010	https://www.shelxle.org/shelx/eingabe.php
HKL2MAP	Pape and Schneider, 2004	https://sbgrid.org/software/titles/hkl2map/
SerialEM	Mastronarde, 2005	https://bio3d.colorado.edu/SerialEM/
MotionCor2	Zheng et al., 2017	https://emcore.ucsf.edu/ucsf-software
Relion	Zivanov et al., 2018	https://www3.mrc-lmb.cam.ac.uk/relion/index.php?title=Main_Page
CTFFIND4	Rohou and Grigorieff, 2015	https://grigoriefflab.umassmed.edu/ctffind4
Servalcat	Yamashita et al., 2021	https://github.com/keitaroyam/servalcat
COOT	Emsley and Cowtan, 2004; Nicholls et al., 2014	https://www2.mrc-lmb.cam.ac.uk/personal/pemsley/coot/
PHENIX	Afonine et al., 2018	https://www.phenix-online.org/
MolProbity	Chen et al., 2010	https://www.phenix-online.org/documentation/reference/molprobity_tool.html
UCSF-ChimeraX	Pettersen et al., 2021	https://www.rbvi.ucsf.edu/chimerax/
CueMol	N/A	http://www.cuemol.org
Larch Python library	10.1088/1742-6596/430/1/012007	https://xraypy.github.io/xraylarch/
VMD	Humphrey, 1996	http://www.ks.uiuc.edu/Research/vmd/
NAMD 2.13	Phillips et al., 2005	http://www.ks.uiuc.edu/Research/namd/
**Other**		
Amicon Ultra-4 Centrifugal Filter Units -10,000 NMWL	Millipore	UFC801024
Ni-NTA Superflow	QIAGEN	30450
HiTrap SP HP	GE Healthcare	17115201
Superdex 200 Increase 10/300	GE Healthcare	28990944
HiLoad 16/600 Superdex 200	GE Healthcare	28989335
5′-Cy5-labeled target RNA	Eurofins	N/A
300 mesh R 1.2/1.3 holey carbon Au	Quantifoil	https://www.quantifoil.com/products/quantifoil/quantifoil-circular-holes/

### Resource Availability

#### Lead contact

Further information and requests for resources and reagents should be directed to and will be fulfilled by the Lead Contact, Osamu Nureki (nureki@bs.s.u-tokyo.ac.jp).

#### Experimental Model and Subject Details

*Escherichia coli* cells were cultured at 37°C in LB medium (containing 20 mg/l kanamycin) for plasmid and protein preparation. HEK293FT cells were grown at 37°C in Dulbecco’s Modified Eagle Medium with high glucose, sodium pyruvate, and GlutaMAX (Thermo Fisher Scientific), supplemented with 1 × penicillin–streptomycin (Thermo Fisher Scientific), 10 mM HEPES (Thermo Fisher Scientific), and 10% fetal bovine serum (VWR Seradigm).

#### Data and code availability

- The atomic models have been deposited in the Protein Data Bank under the accession codes 6VTI (binary complex) and 6VTN (ternary complex). The cryo-EM density map has been deposited in the Electron Microscopy Data Bank under the accession code EMD-32118 (ternary complex). Raw movies of the cryo-EM dataset have been deposited in the Electron Microscopy Public Image Archive under the accession code EMPIAR-11110. The data of unprocessed image files have been deposited in the Mendeley Data repository (doi: 10.17632/nst3mp7h2t.1).
- This paper does not report original code.
- Any additional information required to reanalyze the data reported in this paper is available from the Lead Contact upon request.

#### Materials availability

All unique/stable reagents generated in this study are available from the Lead Contact with a completed Materials Transfer Agreement.

### Method Details

#### Protein and RNA preparation

The gene encoding Cas13bt3 (also known as Cas13X.1; residues 1–775) was synthesized by GenScript and cloned into the modified pE-SUMO vector (LifeSensors). Mutations were introduced by a PCR-based method, and sequences were confirmed by DNA sequencing (Table S1). The N-terminally His_6_-tagged Cas13bt3 protein was expressed in *E. coli* Rosetta2 (DE3). The *E. coli* cells were cultured at 37°C until the OD_600_ reached 0.8, and protein expression was then induced by the addition of 0.1 mM isopropyl β-D-thiogalactopyranoside (Nacalai Tesque). The *E. coli* cells were further cultured at 20°C overnight, harvested by centrifugation, resuspended in buffer Å (20 mM Tris-HCl, pH 8.0, 20 mM imidazole, 1 M NaCl, and 1 mM DTT), and then lysed by sonication. The lysates were centrifuged, and the supernatant was mixed with 3 mL Ni-NTA Superflow resin (QIAGEN). The mixture was loaded into a Poly-Prep Column (Bio-Rad), and the protein was eluted with buffer B (20 mM Tris-HCl, pH 8.0, 0.3 M imidazole, 0.3 M NaCl, and 1 mM DTT). The protein was incubated with TEV protease overnight, and then loaded onto a 5 mL HiTrap SP column (GE Healthcare), equilibrated with buffer C (20mM Tris-HCl, pH 8.0, 0.3 M NaCl, and 1 mM DTT). The protein was eluted with a linear gradient of 0.3–2 M NaCl, and then further purified on a Hiload Superdex 200 column (GE Healthcare), equilibrated with buffer D (20 mM Tris-HCl, pH 8.0, 0.5 M NaCl, and 1 mM DTT). The purified protein was stored at –80°C until use. For structural studies, the crRNA and the target RNA were purchased from Ajinomoto Bio-Pharma. For *in vitro* RNA cleavage experiments, the crRNAs and the target RNA were transcribed *in vitro* with T7 RNA polymerase, and purified by 10% denaturing (7 M urea) polyacrylamide gel electrophoresis (PAGE) (Table S1).

#### Crystallography

The Cas13bt3-crRNA binary complex was reconstituted by mixing the purified dCas13bt3 (R84A/H89A/R739A/H744A) and the 41-nucleotide crRNA at a molar ratio of 1:1.2. The binary complex was purified on a Superdex 200 Increase 10/300 column (GE Healthcare), equilibrated with buffer E (20 mM Tris-HCl, pH 8.0, 150 mM NaCl, 5 mM MgCl_2_, and 1 mM DTT). The purified complex was crystallized at 20°C by the hanging-drop vapor diffusion method. Crystals were obtained by mixing 1 μL of complex solution (A_260 nm_ = 15) and 1 μL of reservoir solution (15% PEG 3,350 and 0.2 M sodium bromide). The SeMet-labeled protein was crystallized under conditions similar to those for the native protein. X-ray diffraction data of the SeMet-labeled protein were collected at 100 K using an EIGER X16M detector (Dectris) and a wavelength of 0.9780 Å on beamline BL41XU at SPring-8 (Hyogo, Japan). The crystals were cryoprotected in reservoir solution supplemented with 25% ethylene glycol. X-ray diffraction data were processed using KAMO (Yamashita et al., 2018) and XDS (Kabsch et al., 2010). The structure was determined by the Se-SAD method, using SHELXC/D/E (Sheldrick, 2010) and HKL2MAP (Pape and Schneider, 2004).

#### Cryo-EM analysis

The Cas13bt3=crRNA-target RNA ternary complex was reconstituted by mixing the purified dCas13bt3 (R84A/H89A/R739A/H744A), the 61-nucleotide crRNA, and the 25-nucleotide target RNA at a molar ratio of 1:1.2:1.4. The ternary complex was purified on a Superdex 200 Increase 10/300 column, equilibrated with buffer F (20 mM Tris-HCl, pH 8.0, 25 mM NaCl, 5 mM MgCl_2_, and 1 mM DTT). The purified complex solution (A_260 nm_ = 6) was applied to freshly glow-discharged Au/Rh 300 mesh R1.2/1.3 grids (Quantifoil) on both sides, using a Vitrobot Mark IV (FEI) at 4°C, with a waiting time of 10 s and a blotting time of 4 s under 100% humidity conditions. The grids were plunge-frozen in liquid ethane cooled at liquid nitrogen temperature.

Cryo-EM data were collected using a Titan Krios G3i microscope (Thermo Fisher Scientific), running at 300 kV and equipped with a Gatan Quantum-LS Energy Filter (GIF) and a Gatan K3 Summit direct electron detector in the electron counting mode (The University of Tokyo, Japan). Movies were recorded at a nominal magnification of 105,000×, corresponding to a calibrated pixel size of 0.83 Å at the electron exposure of 13 e^–^/pix/sec for 2.6 s, resulting in an accumulated exposure of 48.0 e^–^/Å^2^. In total, 2,772 movies were automatically acquired by the image shift method using the SerialEM software (Mastronarde, 2005), with a defocus range of –0.8 to –1.6 μm. The dose-fractionated movies were subjected to beam-induced motion correction and dose-weighting, using MotionCor2 (Zheng et al., 2017) implemented in RELION-3 (Zivanov et al., 2018), and the contrast transfer function (CTF) parameters were estimated using CTFFIND4 (Rohou and Grigorieff, 2015).

Data were processed using RELION-3.1. From the 2,727 motion-corrected and dose-weighted micrographs, 2,380,559 particles were initially picked and extracted at a pixel size of 3.26 Å. These particles were subjected to several rounds of 2D and 3D classifications. The 582,042 best selected particles were then re-extracted at a pixel size of 1.22 Å and subjected to 3D refinement. The resulting 3D model and particle set were subjected to per-particle defocus refinement, beam-tilt refinement, and Bayesian polishing (Zivanov et al., 2020). The 3D refinement and postprocessing of this class yielded a map with global resolutions of 3.20 Å (Cas13b-t3_overall_), according to the Fourier shell correlation (FSC) = 0.143 criterion (Rosenthal and Henderson, 2003). Whereas clear densities were observed for the REC lobe, the NUC lobe was not well resolved in the density map. The no-align 3D classification using a mask covering the NUC lobe resulted in three different classes (classes 1–3). The NUC lobe was well resolved in the class 3 density map, as compared to those in classes 1 and 2. The final refinement and postprocessing of class 3 yielded a map with a global resolution of 3.38 Å (Cas13bt3_NUC_), and this map was used for modeling. The local resolution was estimated by RELION-3.1.

#### Model building and validation

The model of the binary complex was automatically built using Buccaneer (Cowtan, 2006) and Nautilus (Cowtan, 2012), followed by manual model building using COOT (Emsley and Cowtan, 2004) and structural refinement using the SAD function implemented in REFMAC5 (Skubák et al., 2004). The model of the ternary complex was built using the model of the binary complex as a reference, and refined using phenix.real_space_refine ver. 1.16 (Afonine et al., 2018), with secondary structure and base pair/stacking restraints. The model was finally refined against unsharpened half maps using REFMAC5 in the Servalcat pipeline (Yamashita et al.,2021), using secondary structure restraints prepared by ProSMART (Nicholls et al., 2014) and LIBG (Brown et al., 2015). The structure validation was performed using MolProbity (Chen et al., 2010) in the PHENIX package. Residues 1–13, 225–237, and 271–284 of Cas13bt3, and nucleotide 5 of the crRNA are not included in the final model of the binary complex, while residues 1–13, 222–237, 269–287, 626–634, and 703–729 of Cas13bt3, nucleotides 21–25 of the crRNA, and nucleotides 21–25 of the target RNA are not included in the final model of the ternary complex, due to the lack of clear densities. Molecular graphics figures were prepared using UCSF ChimeraX (Pettersen et al., 2021), PyMOL (http://www.pymol.org) and CueMol (http://www.cuemol.org).

#### Phylogenetic analysis

Cas13bt and related Cas13b sequences were retrieved from the NCBI, using a psi-blast search with Cas13bt1 as the query sequence on the HH-suite NR90 database with an E-value reporting and gathering threshold of 1e-3 (Zimmermann et al., 2018). Sequences from PDB structures were removed, because PDB sequences often contain various unnatural modifications and truncations, such as N-terminal His tags. The resulting sequences were then aligned using MAFFT-einsi (Katoh and Standley, 2013). PbuCas13b and previously reported Cas13bts were then added into the set as references, and the full set was deduplicated with priority given to the reference sequences. Partial protein sequences without coverage in both the conserved N-terminal and C-terminal regions of the alignment were then removed (using coverage to the NXAXXN and RNXXXH motifs, respectively). The remaining sequences were then realigned using MAFFT-einsi and columns with gap proportions larger than 0.5 were removed. The resulting trimmed alignment was then used to create a phylogenetic tree with IQ-Tree 2 (Minh et al., 2020). First, modeltest was used to identify the VT+F+I+G4 substitution model as optimal, according to both the AIC and BIC scores (Kalyaanamoorthy et al., 2017). IQ-Tree 2 was then used to construct a tree using this model with 5000 ultrafast bootstraps, 500 initial trees, 100 top initial trees, and maintenance of the best 20 trees along the search, along with the bnni option, which optimizes each bootstrap tree using NNI on the bootstrap alignment (Hoang et al., 2018). The resulting tree was then inspected for high bootstrap values for confident branching points between Cas13bt clades as Cas13b. The presence of two Cas13bt groups separated by Cas13bs with high bootstrap values indicated that Cas13bt, in relation to Cas13b, is not monophyletic.

#### *In vitro* RNA cleavage assay

The RNA cleavage activity of Cas13bt3 was measured *in vitro*, using a 5′-Cy5-labeled target RNA containing a 30-nucleotide spacer-complementary sequence (Table S1). The Cas13bt3-crRNA complex was prepared by mixing the purified Cas13bt3 (2 μM) and the crRNA (3 μM) at 37°C for 5 min. The pre-assembled Cas13bt3–crRNA complex (5 μL, 200 nM final concentration) was mixed with the target RNA (15 μL, 200 ng), and then incubated at 37°C for 0.5,15, 30 or 60 min in 20 μL reaction buffer, containing 20 mM HEPES-NaOH, pH 7.5, 100 mM KCl, 2 mM MgCl_2_,1 mM DTT, and 5% glycerol. The reaction was stopped by the addition of quench buffer, containing EDTA (20 mM final concentration) and Proteinase K (40 ng). The reaction products were fractionated by 10% denaturing (7 M urea) PAGE, and then visualized using an Amersham Imager 600 (GE Healthcare). *In vitro* RNA cleavage experiments were performed at least three times.

#### *In vitro* processing assay

The pre-crRNA processing was examined *in vitro*, using the purified Cas13bt3 and its pre-crRNA containing two spacer-DR units. The Cas13bt3 protein (8 μL, 800 nM final concentration) was mixed with the pre-crRNA (12 μL, 200 nM final concentration), and then incubated at 37°C for 2 h in 20 μL processing buffer, containing 20 mM HEPES-NaOH, pH 7.5, 50 mM NaCl, and 0.5 mM MgCl_2_. The reaction was stopped by the addition of quench buffer. The reaction products were then fractionated by 10% denaturing (7 M urea) PAGE. The gels were stained with SYBR Gold Nucleic Acid Gel Stain (Thermo Fisher Scientific). The purified PbuCas13b and its pre-crRNA were used as the positive control. *In vitro* processing experiments were performed at least three times.

#### MD simulation

The simulation system included the Cas13bt3-crRNA binary complex or Cas13bt3-crRNA-target RNA ternary complex, TIP3P water, and 150 mM NaCl. Disordered regions of Cas13bt3 in the binary and ternary complexes were modeled using MODELLER (Šali and Blundell, 1993), and missing hydrogen atoms were built using VMD (Humphrey et al., 1996). The net charge of the simulation system was neutralized by the addition of 150 mM NaCl. The simulation systems were 120 × 144 × 120 Å^3^, and contained 197,387 and 196,537 atoms in the binary and ternary complexes, respectively. The molecular topologies and parameters from the Charmm36 force field (Best et al., 2012; Klauda et al., 2010) were used for the protein, RNA, and water molecules. Molecular dynamics simulations were performed with the program NAMD 2.13 (Phillips et al., 2005). The simulation systems were energy minimized for 1,000 steps with fixed positions of the non-hydrogen atoms. After minimization, another 1,000 steps of energy minimization were performed with 10 kcal mol^–1^ restraints for the non-hydrogen atoms. Next, equilibrations were performed for 0.1 ns under NVT conditions, with 10 kcal mol^–1^ Å^–2^ restraints for heavy atoms in the protein. Finally, equilibration was performed for 2.0 ns under NPT conditions, with 1.0 kcal mol ^–1^ Å^–2^ restraints for all Cα atoms of the protein. The production runs were performed for 200 ns without restraints, while maintaining constant temperature at 310 K using Langevin dynamics and constant pressure at 1 atm using a Nosé-Hoover Langevin piston (Feller et al., 1995). The long-range electrostatic interactions were calculated by the particle mesh Ewald method (Darden et al., 1993). The simulation results were analyzed and visualized with mdtraj (McGibbon et al., 2015), Seaborn, and CueMol.

#### Mammalian cell culture and transfection

Mammalian cell culture experiments were performed with the HEK293FT line (American Type Culture Collection (ATCC)) grown in Dulbecco’s Modified Eagle Medium with high glucose, sodium pyruvate, and GlutaMAX (Thermo Fisher Scientific), supplemented with 1 × penicillin–streptomycin (Thermo Fisher Scientific), 10 mM HEPES (Thermo Fisher Scientific), and 10% fetal bovine serum (VWR Seradigm). All cells were maintained at confluency below 80%. All transfections were performed with Lipofectamine 2000 (Thermo Fisher Scientific) in 96-well plates unless otherwise noted. Cells were plated at 2×10^4^ cells/well 16–20 h prior to transfection, to ensure 90% confluency at the time of transfection. For each well on the plate, transfection plasmids were combined with OptiMEM I Reduced Serum Medium (Thermo Fisher Scientific) to a total of 25μL. Separately, 23 μL of OptiMEM was combined with 2 μL of Lipofectamine 2000. The plasmid and Lipofectamine solutions were then combined and pipetted onto cells.

#### Mammalian RNA knockdown assays

HEK293FT cells were transfected as described with 25 ng of a plasmid encoding the Cas13bt3 variant expressed from a CMV promoter, 300 ng of a plasmid encoding a crRNA expressed from a human U6 promoter, and 45 ng of a dual *Gaussia/Cypridina(W85X)* luciferase reporter plasmid (Cox et al., 2017) (Table S1). After 48 h, the culture media were aspirated from the cell samples and the *Cypridina* and *Gaussia* luciferase activities in the media were measured using *Gaussia* and *Cypridina* Luciferase Assay Kits (Targeting Systems) with the injection protocol on a Biotek Synergy Neo 2 (Agilent) imaging reader. Each experimental luciferase measurement was normalized to the appropriate control luciferase measurement *(i.e*., if the *Cypridina* luciferase was targeted, then the *Gaussia* luciferase measurement was used as the control value and *vice versa)*.

#### Mammalian RNA editing assays

HEK293FT cells were transfected as described with 25 ng of a plasmid encoding a dCas13bt3-ADAR2dd(E488Q) fusion expressed from a CMV promoter, 300 ng of a plasmid encoding a crRNA expressed from a human U6 promoter, and 45 ng of a dual *Gaussia/ Cypridina(W85X)* luciferase reporter plasmid (Cox et al., 2017) (Table S1). After 48 h, the RNA was harvested, and reverse transcription was then performed as described (Joung et al., 2017), using a gene-specific primer for the *Cypridian* luciferase (5′-TTTGCATTCATCTGGTACTTCTAGGGTGTC-3′). cDNA was used as input for the preparation of next-generation sequencing libraries with NEBNext High-Fidelity 2 × PCR Master Mix (NEB), and amplicons were then sequenced on an Illumina MiSeq. Editing was quantified by counting the number of reads at which the expected edited position in the amplicon was identified as G and dividing by the total number of reads in the sample, using Python. Unless otherwise noted, all reported data are the average of four biological replicates. To measure the restoration of the *Cypridina* luciferase (W85X) activity, the culture media were aspirated from the same cell samples and the *Cypridina* and *Gaussia* luciferase activities in the media were measured as described above.

#### Quantification and Statistical Analysis

*In vitro* experiments were performed at least three times.

## Supplementary Material

Article plus Supplementary Information

Figures S1-S7 & Table S1

## Figures and Tables

**Table 1:** Crystallographic data collection, model refinement, and validation

**Data collection and processing**	
Sample	Cas13bt3-crRNA
PDB ID	7VTI
Beamline	SPring-8 BL41XU
Wavelength (Å)	0.9780
Space group	*P*2_1_2_1_2_1_
**Cell dimensions**	
a, b, c (Å)	78.50, 95.52, 125.45
α, β, γ (°)	90, 90, 90
Resolution (Å)	50–1.89(2.00–1.89)
R_meas_^a^	0.133(2.99)
*I*/σ*/*	14.59(0.93)
CC(1/2)^a^	0.999 (0.520)
Completeness (%)^a^	0.999 (0.993)
Multiplicity^a^	13.98 (13.89)
**Refinement**	
No. of reflections	73,944
R_work_/R_free_	0.197/0.243
No. of atoms	
Protein	6,075
Nucleic acid	904
Others	436
*B*-factors (Å)	
Protein	49.7
Nucleic acid	41.6
Others	49.9
RMSDs	
Bond lengths (Å)	0.011
Bond angles (°)	1.78
Ramachandran plot	
Favored (%)	97.26
Allowed (%)	2.74
Outliers (%)	0.00

a Friedel pairs are treated as different reflections.

**Table 2:** Cryo-EM data collection, model refinement, and validation

**Data collection and processing**	
Sample	Cas13bt3-crRNA-target RNA
EMDB ID	EMD-32118
PDB ID	7VTN
Microscope	Titan Krios G3i
Detector	Gatan K3 camera
Magnification	105,000
Voltage (kV)	300
Electron exposure (e^–^/Å^2^)	48.0
Defocus range (μm)	–0.8 to –1.6
Pixel size (Å)	0.83
Symmetry imposed	C1
Number of movies	2,772
Initial particle images (no.)	2,380,559
Final particle images (no.)	105,869
Map resolution (Å)	3.4
FSC threshold	0.143
Map-sharpening *B* factor (Å^2^)	–110.1
**Refinement**	
No. of atoms	
Protein	5,688
Nucleic acid	1,617
R.m.s. deviations	
Bond lengths (Å)	0.008
Bond angles (°)	1.50
**Validation**	
Clashscore	2.10
Rotamer outliers (%)	3.19
Ramachandran plot	
Favored (%)	96.63
Allowed (%)	3.08
Outliers (%)	0.29

## Highlights

- Cas13bt3-crRNA binary complex and Cas13bt3-crRNA-target RNA ternary complex structures
- Structural basis for miniaturization of the minimal Cas13bt3 scaffold
- Cas13b activation mechanism is distinct from those of other Cas13a/d family enzymes
- Structure-guided engineering of ultracompact RNA base editors
