# Supplementary material for: Structure and engineering of the minimal type VI CRISPR-Cas13bt3
Source: Mol Cell. Author manuscript; Available in PMC 2022 Oct 11. (PMC7613696; doi:10.1016/j.molcel.2022.08.001)
Supplement: Figures S1-S7 & Table S1 [file EMS155324-supplement-Figures_S1_S7___Table_S1.pdf]

Molecular Cell, Volume *82*

Supplemental information

Structure and engineering of the minimal

type VI CRISPR-Cas13bt3

Ryoya Nakagawa, Soumya Kannan, Han Altae-Tran, Satoru N. Takeda, Atsuhiro Tomita, Hisato Hirano, Tsukasa Kusakizako, Tomohiro Nishizawa, Keitaro Yamashita, Feng Zhang, Hiroshi Nishimasu, and Osamu Nureki

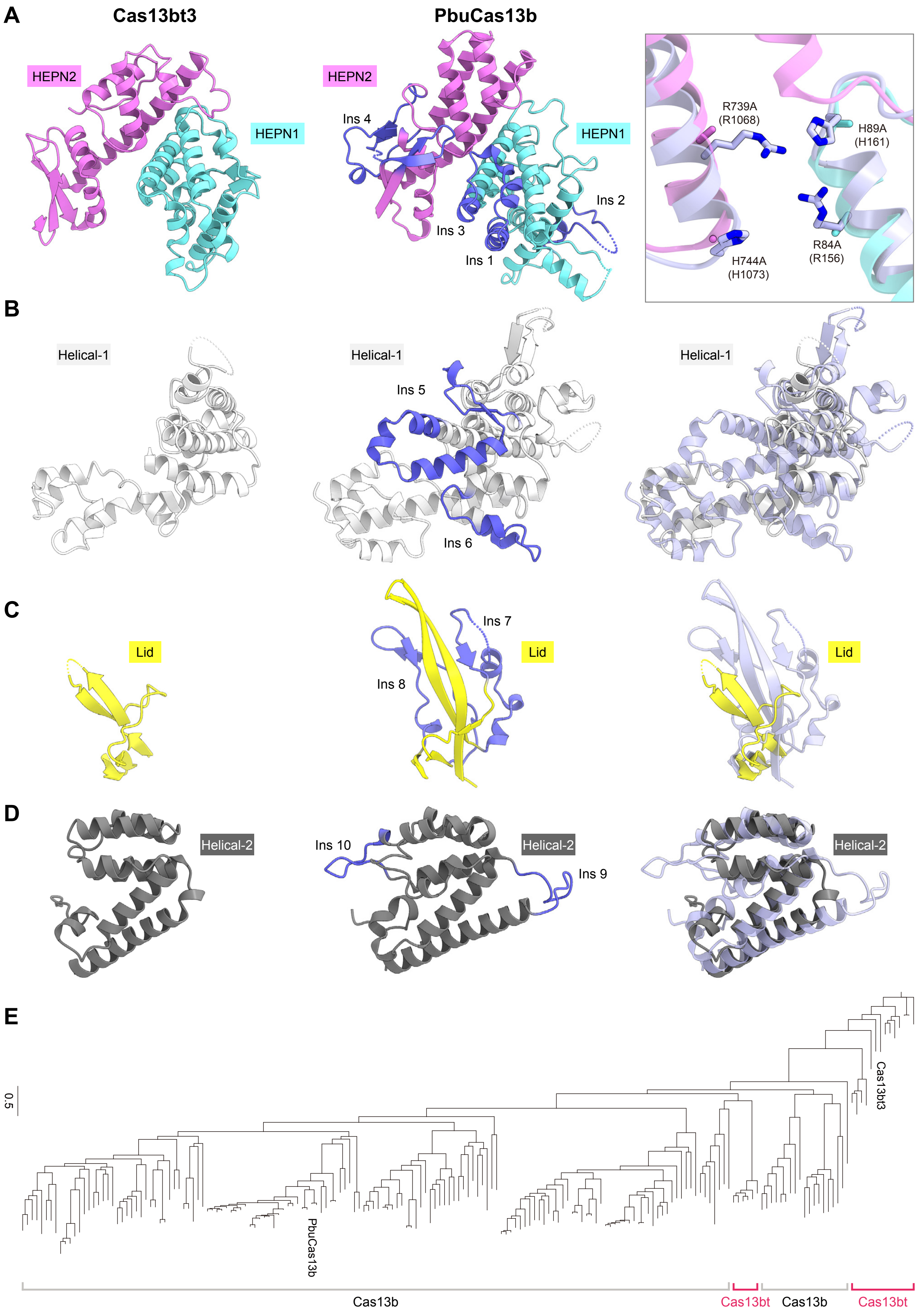

Figure S1. Domain structures, Related to Figure 1

(A–D) Structural comparison of the HEPN (A), Helical-1 (B), Lid (C), and Helical-2 (D) domains of binary complexes of Cas13bt3 and PbuCas13b (PDB: 6DTD). In the PbuCas13b structure, the PbuCas13b-specific insertions are highlighted in blue. PbuCas13b (light blue) is superimposed onto Cas13bt3. (E) Phylogenetic tree of Cas13b and Cas13bt proteins.

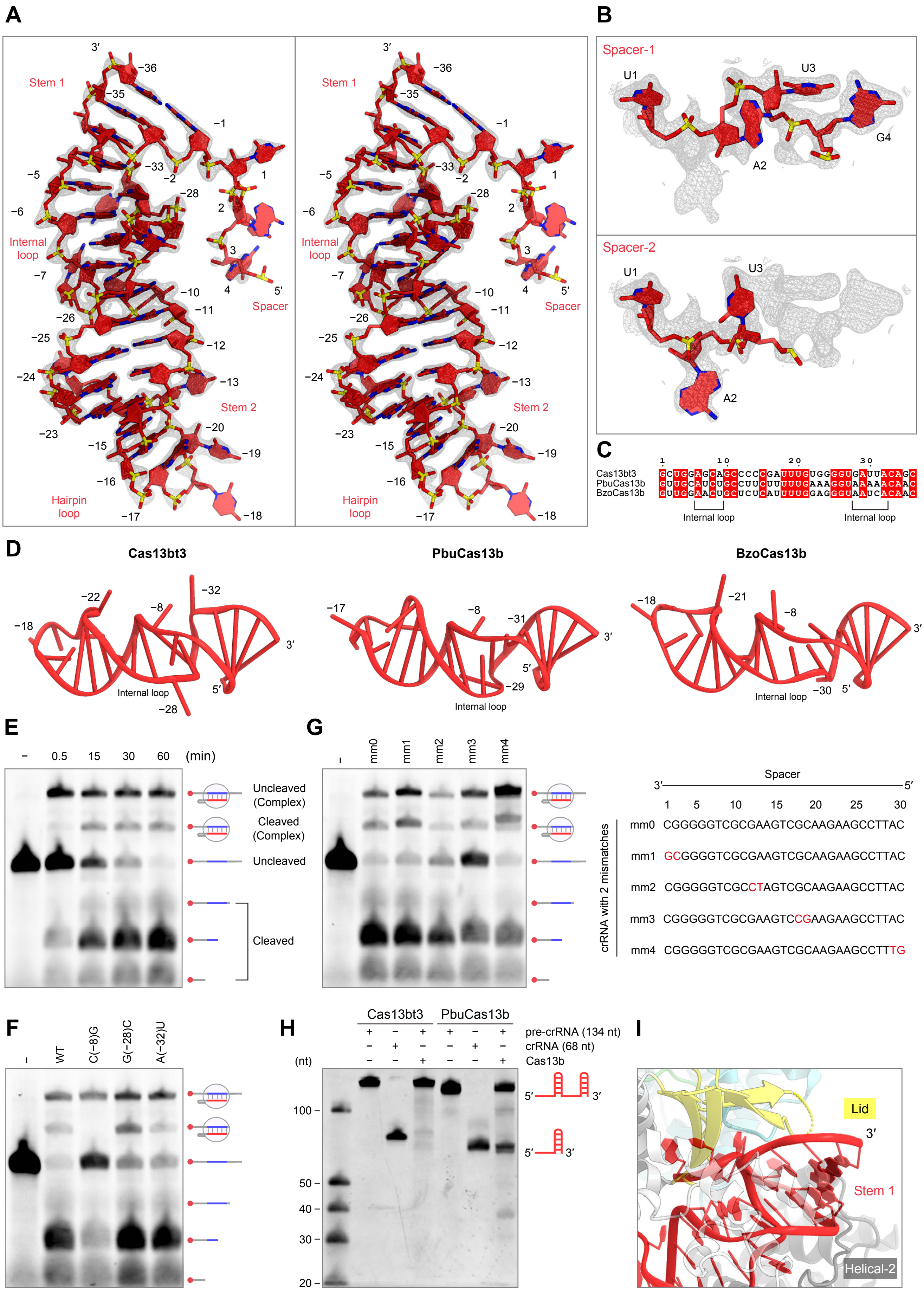

A
3′
−36
Stem 1
−35
−1
−5
−33
−2
1
−28
−6
2
Internal loop
3
−7
−10
4
5′
−11
Spacer
−26
−25
−12
−24
−13
Stem 2
−23
−20
−15
−19
−16
Hairpin loop
−17
−18
B
Spacer-1
U1
U3
G4
A2
Spacer-2
U1
U3
A2
C
Cas13bt3
PbuCas13b
BzoCas13b
Internal loop
Internal loop
D
Cas13bt3
PbuCas13b
BzoCas13b
−22
−32
−18
−8
3′
Internal loop
5′
−28
−17
−8
−31
3′
5′
−29
Internal loop
−18
−21
−8
3′
Internal loop
−30
5′
E
−
0.5
15
30
60
(min)
Uncleaved (Complex)
Cleaved (Complex)
Uncleaved
Cleaved
G
-
mm0
mm1
mm2
mm3
mm4
Spacer
3′
5′
1
5
10
15
20
25
30
crRNA with 2 mismatches
mm0 CGGGGGTCGCGAAGTCGCAAGAAGCCTTAC
mm1 GCGGGGTCGCGAAGTCGCAAGAAGCCTTAC
mm2 CGGGGGTCGCCTAGTCGCAAGAAGCCTTAC
mm3 CGGGGGTCGCGAAGTCCGAAGAAGCCTTAC
mm4 CGGGGGTCGCGAAGTCGCAAGAAGCCTTTG
F
-
WT
C(−8)G
G(−28)C
A(−32)U
H
Cas13bt3
PbuCas13b
pre-crRNA (134 nt)
crRNA (68 nt)
Cas13b
(nt)
100
50
40
30
20
5′
3′
5′
3′
I
Lid
3′
Stem 1
Helical-2

Figure S2. crRNA structures, Related to Figure 2

(A) $2mFo - DFc$ electron density map for the crRNA in the binary complex (contoured at 2.5σ) (stereo view).
(B) $2mFo - DFc$ electron density map for the spacer region in two distinct conformations (contoured at 0.8σ).
(C and D) Sequences (C) and structures (D) of the DR regions of the Cas13bt3, PbuCas13b (Slaymaker et al., 2019) (PDB: 6DTD), and BzoCas13b (Zhang et al., 2018) (PDB: 6AAY) crRNAs. The DR sequence of Cas13bt3 crRNA shares 58% and 67% identity with those of PbuCas13b and BzoCas13b, respectively.
(E) *In vitro* RNA cleavage experiments. The 5′-Cy5-labeled target RNA was incubated with the Cas13bt3–crRNA complex at 37°C for 0.5, 15, 30 and 60 min, and then analyzed by denaturing urea-PAGE.
(F and G) Effects of mutations in the DR (F) and spacer (G) regions of the crRNA. The 5′-Cy5-labeled target RNA was incubated with the Cas13bt3–crRNA complex (wild-type or mutant crRNAs) at 37°C for 60 min, and then analyzed by denaturing urea-PAGE.
(H) *In vitro* processing experiments. The pre-crRNA was incubated with Cas13bt3 at 37°C for 60 min, and then analyzed by denaturing urea-PAGE. PbuCas13b was used as a positive control.
(I) Location of the 3′ end of the crRNA. Disordered regions are indicated as dotted lines.

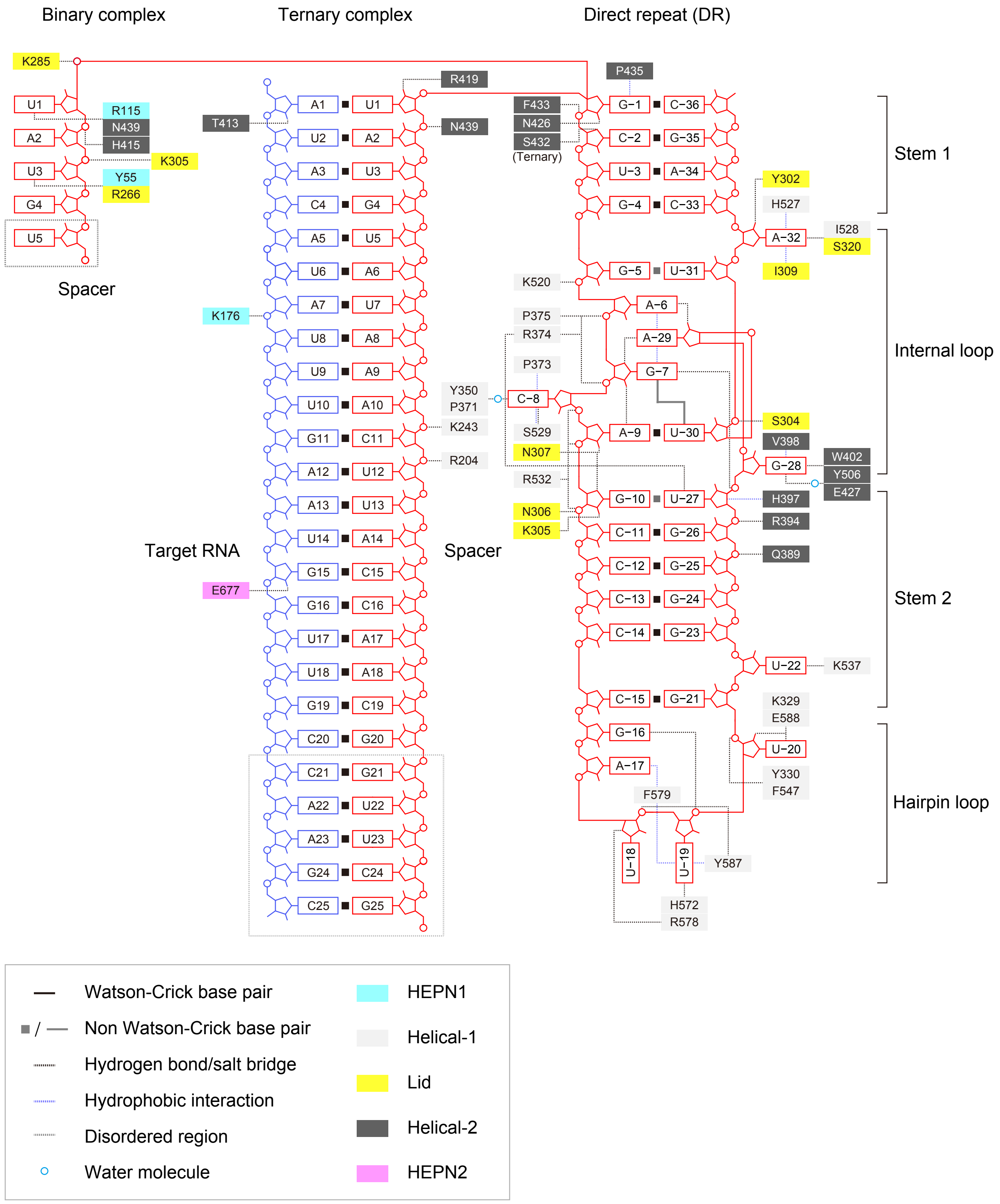

Figure S3. Schematics of RNA recognition, Related to Figures 3 and 5

The disordered regions are boxed by dashed grey lines.

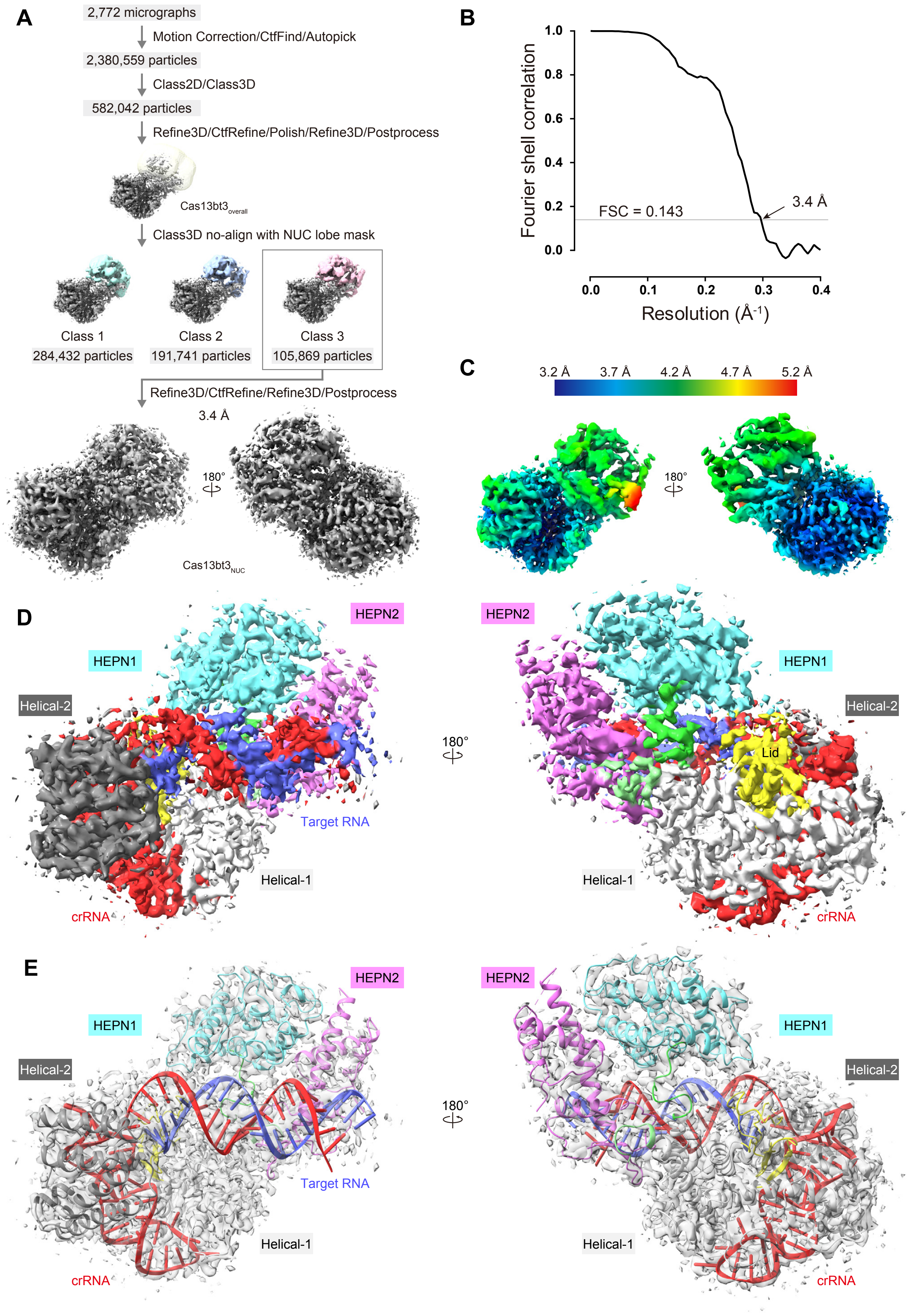

Figure S4. Cryo-EM analysis of the Cas13bt3–crRNA–target RNA ternary complex, Related to Figure 4

(A) Single-particle cryo-EM image processing workflow.

(B) Fourier shell correlation curve for the 3D reconstruction.

(C) Local resolution of the cryo-EM density map.

(D and E) Cryo-EM density maps of the Cas13bt3–crRNA–target RNA ternary complex.

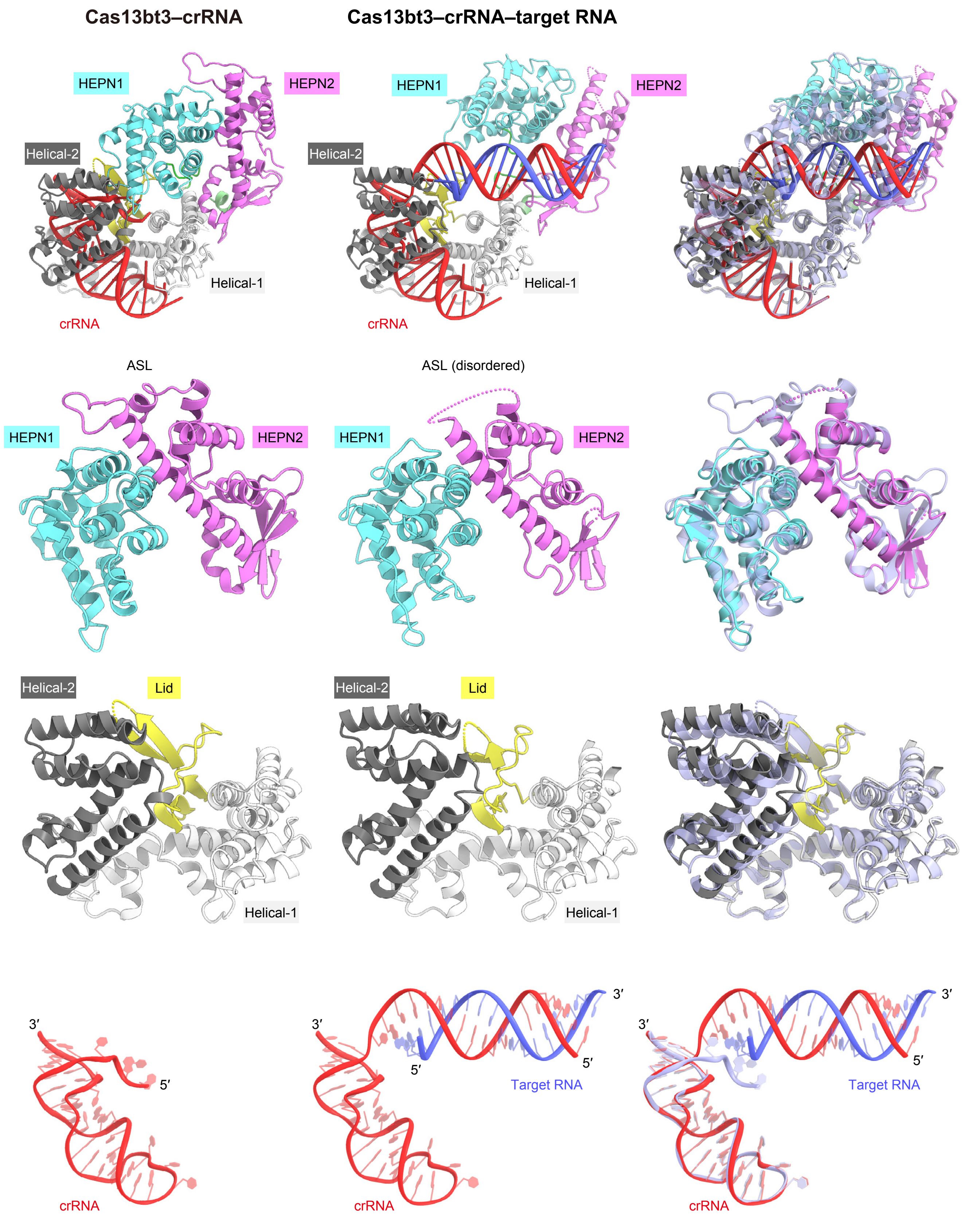

Figure S5. Structural comparison between the Cas13bt3 binary and ternary complexes, Related to Figure 5
Structures of the Cas13bt3–crRNA binary complex (left) and the Cas13bt3–crRNA–target RNA ternary complex (center). The binary complex (light blue) is superimposed onto the ternary complex (right).

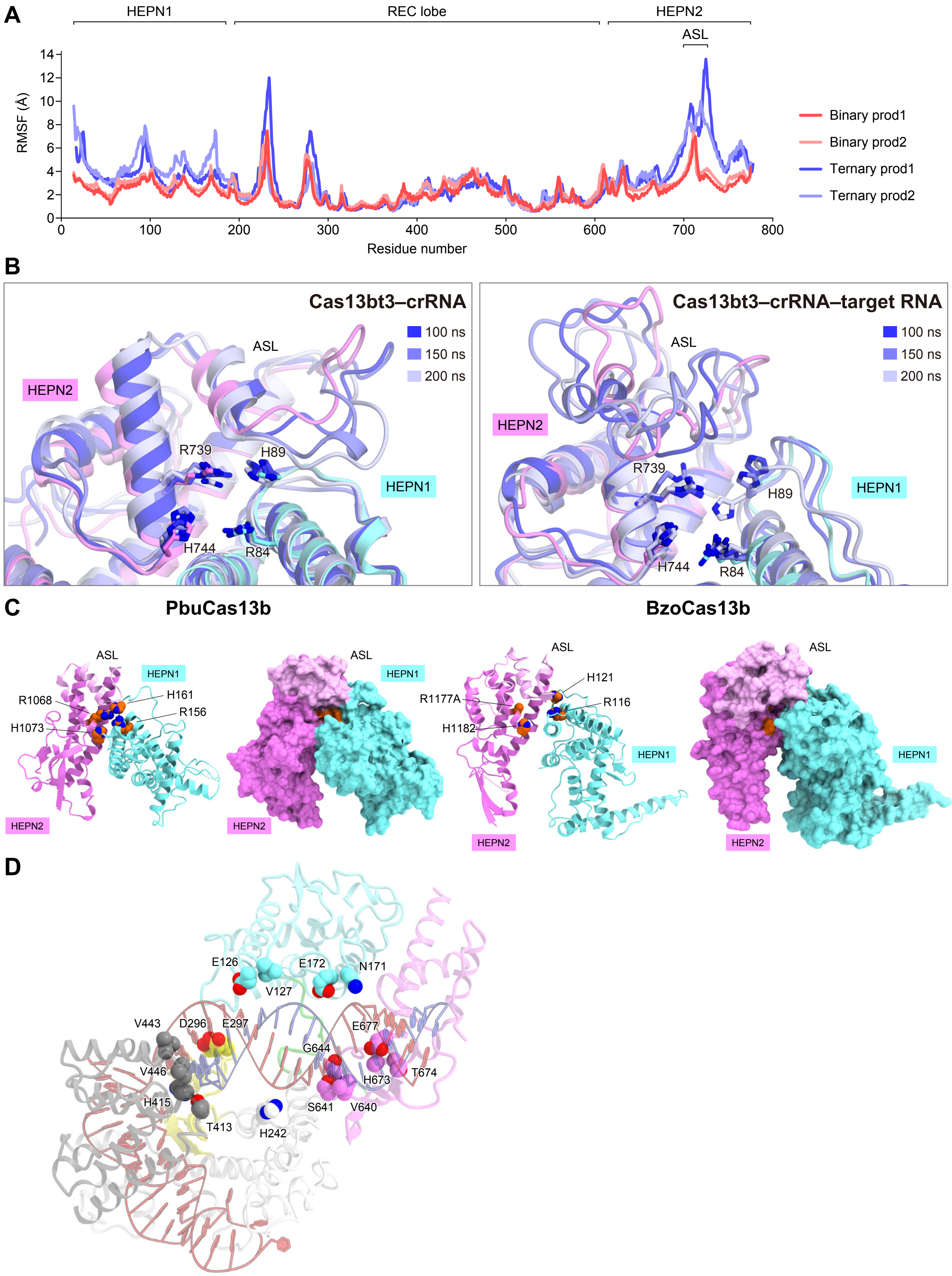

Figure S6. Structural flexibility in the HEPN active site and molecular engineering, Related to Figure 6

(A) MD simulations of the Cas13bt3 binary (red) and ternary (blue) complexes. RMSF values for equivalent Cα atoms were calculated by aligning the structures based on their crRNA DR regions during 200-ns MD simulations.

(B) Superimposition of the HEPN active sites in the binary (left) and ternary (right) complexes after 100-, 150-, and 200-ns MD simulations.

(C) Structures of the HEPN domains of PbuCas13b and BzoCas13b.

(D) Mapping of the 17 residues onto the Cas13bt3–crRNA–target RNA complex. Since mutations, except for the E172R and E297F, did not substantially improve Cas13bt3-mediated RNA cleavage, we focused on the E172R and E297F mutations.

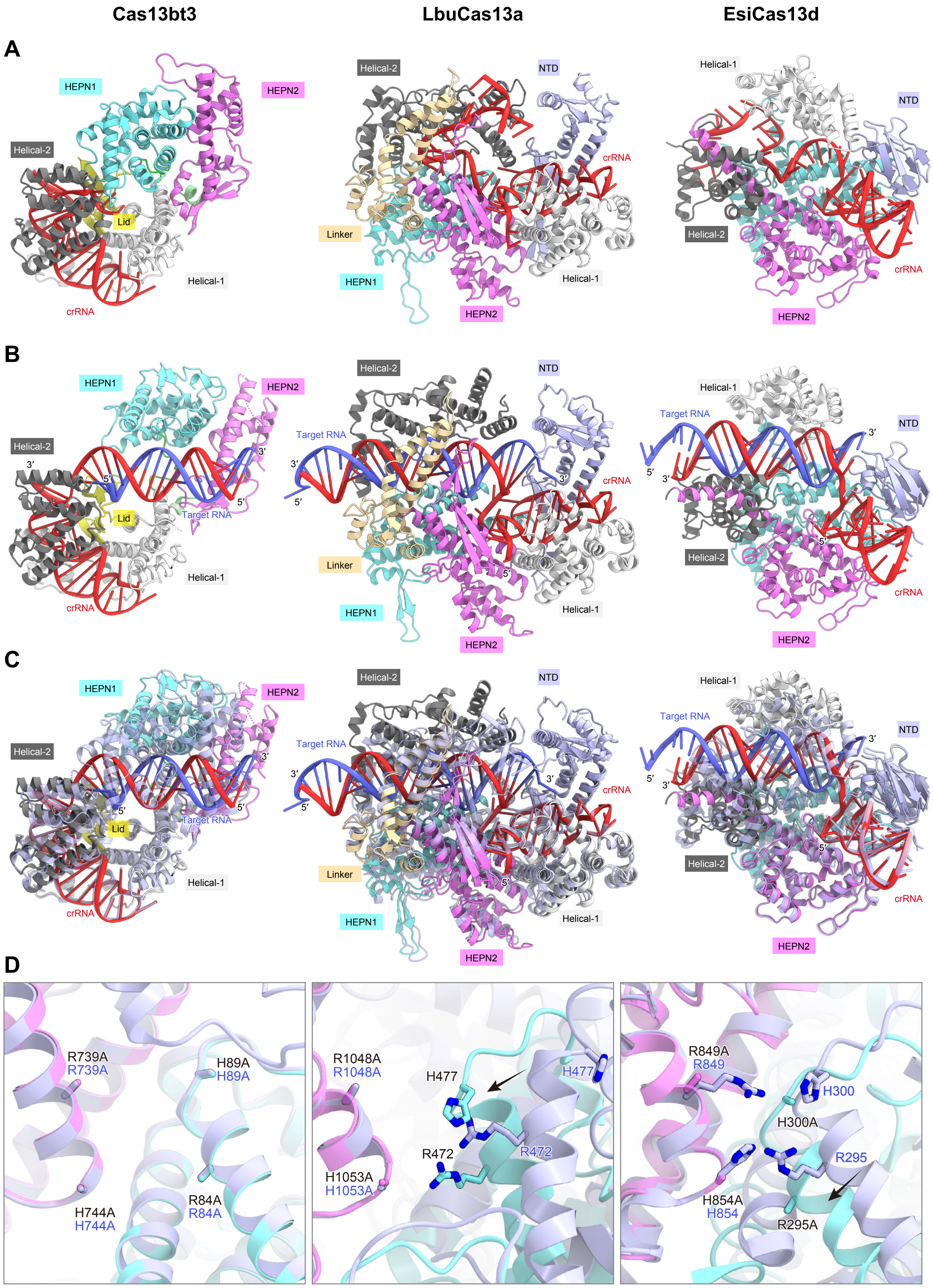

Figure S7. Structural comparisons of Cas13bt3 with LbuCas13a and EsiCas13d, Related to Figure 7

(A) Binary complex structures of Cas13bt3 (left), LbuCas13a (PDB: 5XWY) (center), and EsiCas13d (PDB: 6E9E) (right).

(B) Ternary complex structures of Cas13bt3 (left), LbuCas13a (PDB: 5XWP) (center), and EsiCas13d (PDB: 6E9F) (right).

(C and D) Superimposition of the overall structures (C) and the HEPN active sites (D) in the binary (light blue) and ternary (colored as in A) complexes of Cas13bt3, LbuCas13a, and EsiCas13d.

Table S1. Nucleic-acid sequences used in this study, Related to STAR Methods

Oligonucleotides used to introduce the Cas13bt3 mutations (pE-SUMO-His6-Cas13bt3)

Mutation	Forward primer	Reverse primer
R84A/H89A	TTCAGCgccTACAGACACAGCCCCGGCTG	GTAGTTggcCAGAGCCTCGGCCTTGGC
R739A/H744A	TTCTTTgccCACCACCTGAAGTTCGTGATCGATG	GGCTCTggcCACTTTGTTCACGGCGGTTTTCTC
R122A	gccAGAGAAACCGAAGTGATCATCGAGTTCC	CCTGCACTCGAAGATGGCCC
R123A	gccGAAACCGAAGTGATCATCGAGTTCCC	CCGCCTGCACTCGAAGATGG
R155A	gccAGAGTGCTGGACAGACTGTATGGC	TTCCACAAAGAAGCTGACGAAGAACAC
R156A	gccGTGCTGGACAGACTGTATGGCG	GCGTTCCACAAAGAAGCTGACGAAG
K169A	gccAAGAATGAGGGCCAGTACAAGCTGAC	CAGGCCGGACACGGCGC
K170A	gccAATGAGGGCCAGTACAAGCTGACC	CTTCAGGCCGGACACGGCG
K645A	gccCTGTATGTGCTGGACGACGCC	GCCGTAGTCGGACACGCTGAAC
E172R	cggGGCCAGTACAAGCTGACCCG	ATTCTTCTTCAGGCCGGACACGG
E297F	ttcGACCAGAGCTACTACATCAGCAAGAACAAC	GTCCTTCTTGGAGAAGTCCACCACC

The crRNA and target RNA used for the structure determination

crRNA	GCUUGGCAACCAUUCAAAUAUGUAUGCUGGAGCAGCCCCCGAUUUGUGGGGUGAUUACAGC
Target RNA	AUACAUAUUUGAAUGGUUGCCAAGC

crRNAs and target RNA used for the *in vitro* cleavage and processing experiments

crRNA	ggCAUUCCGAAGAACGCUGAAGCGCUGGGGGCGCUGGAGCAGCCCCCGAUUUGUGGGGUGAUUACAGC
crRNA-C(−8)G	ggCAUUCCGAAGAACGCUGAAGCGCUGGGGGCGCUGGAGgAGCCCCCGAUUUGUGGGGUGAUUACAGC
crRNA-G(−28)C	ggCAUUCCGAAGAACGCUGAAGCGCUGGGGGCGCUGGAGCAGCCCCCGAUUUGUGGGGUcAUUACAGC
crRNA-A(−32)U	ggCAUUCCGAAGAACGCUGAAGCGCUGGGGGCGCUGGAGCAGCCCCCGAUUUGUGGGGUGAUUuCAGC
crRNA-mm1	ggCAUUCCGAAGAACGCUGAAGCGCUGGGGcgGCUGGAGCAGCCCCCGAUUUGUGGGGUGAUUACAGC
crRNA-mm2	ggCAUUCCGAAGAACGCUGAtcCGCUGGGGGCGCUGGAGCAGCCCCCGAUUUGUGGGGUGAUUACAGC
crRNA-mm3	ggCAUUCCGAAGAAgcCUGAAGCGCUGGGGGCGCUGGAGCAGCCCCCGAUUUGUGGGGUGAUUACAGC
crRNA-mm4	gggtUUCCGAAGAACGCUGAAGCGCUGGGGGCGCUGGAGCAGCCCCCGAUUUGUGGGGUGAUUACAGC
Target RNA	AAUUUGCCCCCAGCGCUUCAGCGUUCUUCGGAAUGUCGCG
Pre-crRNA	ggCAUUCCGAAGAACGCUGAAGCGCUGGGGGCGCUGGAGCAGCCCCCGAUUUGUGGGGUGAUUACAGCCAUUCCGA AGAACGCUGAAGCGCUGGGGGCGCUGGAGCAGCCCCCGAUUUGUGGGGUGAUUACAGC
PbuCas13b-crRNA	ggCAUGCCAAUGCGCGACAUUCCGAAGAACGCGUUGCAUCUGCCUUCUUUUUGAAAGGUAAAAACAAC
PbuCas13b-pre-crRNA	ggCAUGCCAAUGCGCGACAUUCCGAAGAACGCGUUGCAUCUGCCUUCUUUUUGAAAGGUAAAAACAACCAUGCCAAU GCGCGACAUUCCGAAGAACGCGUUGCAUCUGCCUUCUUUUUGAAAGGUAAAAACAAC

crRNA used for mammalian RNA knockdown and editing assays

Gaussia luciferase crRNA 1	GGGCAUUGGCUUCCAUCUCUUUGAGCACCUGCUGGAGCAGCCCCCGAUUUGUGGGGUGAUUACAGC
Gaussia luciferase crRNA 2	GGAAUGUCGACGAUCGCCUCGCCUAUGCCGGCUGGAGCAGCCCCCGAUUUGUGGGGUGAUUACAGC
Cypridina luciferase X95W crRNA	UUCUAAACCAUCCUGCGGCCUCUACUCUGCGCUGGAGCAGCCCCCGAUUUGUGGGGUGAUUACAGC
Non-targeting crRNA	GUAAUGCCUGGCUUGUCGACGCAUAGUCUGGCUGGAGCAGCCCCCGAUUUGUGGGGUGAUUACAGC

The codons for the mutations and the 5′ GG for *in vitro* transcription are indicated with lower case letters. The guide/target sequences are underlined.
